# Recent Insights into Endogenous Mammalian Cardiac Regeneration Post-Myocardial Infarction

**DOI:** 10.3390/ijms252111747

**Published:** 2024-11-01

**Authors:** Erika Fiorino, Daniela Rossin, Roberto Vanni, Matteo Aubry, Claudia Giachino, Raffaella Rastaldo

**Affiliations:** Department of Clinical and Biological Sciences, University of Turin, Regione Gonzole 10, 10043 Orbassano, Italy; erika.fiorino@unito.it (E.F.); d.rossin@unito.it (D.R.); roberto.vanni@unito.it (R.V.); matteo.aubry@unito.it (M.A.); claudia.giachino@unito.it (C.G.)

**Keywords:** Myocardial infarction, cardiac regeneration, cardiomyocytes, endothelial cells, stem/progenitor cells, angiogenesis, tissue repair, regenerative capacity

## Abstract

Myocardial infarction (MI) is a critical global health issue and a leading cause of heart failure. Indeed, while neonatal mammals can regenerate cardiac tissue mainly through cardiomyocyte proliferation, this ability is lost shortly after birth, resulting in the adult heart’s inability to regenerate after injury effectively. In adult mammals, the adverse cardiac remodelling, which compensates for the loss of cardiac cells, impairs cardiac function due to the non-contractile nature of fibrotic tissue. Moreover, the neovascularisation after MI is inadequate to restore blood flow to the infarcted myocardium. This review aims to synthesise the most recent insights into the molecular and cellular players involved in endogenous myocardial and vascular regeneration, facilitating the identification of mechanisms that could be targeted to trigger cardiac regeneration, reduce fibrosis, and improve functional recovery post-MI. Reprogramming adult cardiomyocytes to regain their proliferative potential, along with the modulation of target cells responsible for neovascularisation, represents promising therapeutic strategies. An updated overview of endogenous mechanisms that regulate both myocardial and coronary vasculature regeneration—including stem and progenitor cells, growth factors, cell cycle regulators, and key signalling pathways—could help identify new critical intervention points for therapeutic applications.

## 1. Introduction

### 1.1. Main Causes and Consequences of Myocardial Infarction

Cardiovascular disease represents the leading cause of death worldwide, partly due to the limited capacity of the adult mammalian heart to regenerate [1]. The heart is the first organ formed during embryonic development and is composed not only of contractile cardiomyocytes (CMs) but also of non-CMs, including endothelial cells (ECs), fibroblasts (FBs), vascular smooth muscle cells (SMCs), immune cells, adipocytes, and neuronal cells, all of which contribute to supporting cardiac function and homeostasis [2]. Myocardial infarction (MI), commonly known as a heart attack, is a serious medical condition that occurs when blood flow to a part of the heart is blocked or markedly reduced for an extended period, causing damage or death of cardiac muscle tissue [3]. While timely reperfusion of the infarct-related coronary artery by percutaneous coronary intervention is life-saving, it may inflict some additional damage to the myocardium, so that final infarct size is determined both by ischaemia and reperfusion-induced (I/R) injury [4].

The consequences of MI can be severe and life-threatening. The damage’s extent and location determine the infarction’s severity and its consequences [5]. The main cause of MI is atherosclerosis, a condition characterised by the accumulation of plaques composed of cholesterol, fatty substances, calcium, and fibrin (a clotting material) within the walls of the coronary arteries [6]. Over time, these plaques can become unstable, leading to rupture or erosion of the plaque surface. When one of these phenomena occurs, platelets in the blood adhere to the injury site, forming a blood clot that can partially or completely block the artery, causing an MI [7]. Several factors influence the development of atherosclerosis and subsequent MI. Among these, high levels of low-density lipoprotein (LDL) cholesterol, hypertension, smoking, diabetes (particularly type 2 diabetes), obesity and a sedentary lifestyle, and, lastly, genetic factors, can lead to the buildup of plaques in the arteries, narrowing the passages and increasing the risk of clot formation [8].

The first event following an MI is the death of millions of myocytes, which triggers the activation of the innate immune response, resulting in tissue infiltration by leukocytes [5]. Neutrophils and macrophages are primarily responsible for extracellular matrix (ECM) destruction. After 1 or 2 days, the tissue reaches the peak of the inflammatory response. Transforming growth factor-beta (TGF-β), one of the main cytokines present in the damaged myocardium, promotes the differentiation of FBs into myofibroblasts, which, together with macrophages, start the remodelling of the cardiac tissue with excessive collagen deposition and fibrosis and the formation of scar tissue within 3–7 days [9]. Following this phase, there is a window of approximately 1–2 months during which the scar matures. These processes collectively work to repair the damage and reshape the tissue, and, unlike many other organs, in cardiac tissue, it is improbable to achieve the “restitutio ad integrum” after injury because of its limited regenerative capacity [10]. The cardiac remodelling initially acts as a compensatory response that guarantees the integrity of the ventricular wall; the persistence of this process displays deleterious long-term haemodynamic consequences because it compromises the contractile activity of the heart due to the rigidity of the scar tissue itself, thus leading to heart failure (HF) development [11]. The progression of this adverse remodelling, i.e., the long-term alteration of cardiac function leads to a volume overload-induced ventricular dilation, is associated with a patient’s poor prognosis [12]. In some cases, MI can lead to complications such as arrhythmias, which are generally due to the alteration of impulse propagation caused by collagen deposition that results in intramyocardial re-entry, which can lead to fatal tachyarrhythmias in the first month after the ischaemic event, with a high risk of sudden cardiac arrest [13].

The long-term consequences of MI can include an increased risk of recurrent heart attacks and reduced quality of life. Cardiac rehabilitation, lifestyle modifications (such as smoking cessation, a healthy diet, regular exercise, and weight control), medications (such as antiplatelet drugs, statins, beta-blockers, and ACE inhibitors), and, in some cases, surgical interventions (such as coronary artery bypass grafting or angioplasty with stent placement) are essential components of management and prevention strategies aimed at reducing the risk of recurrent MI and improving overall cardiovascular health [14,15].

### 1.2. Concepts of Cardiac Regeneration

**Figure 1:**
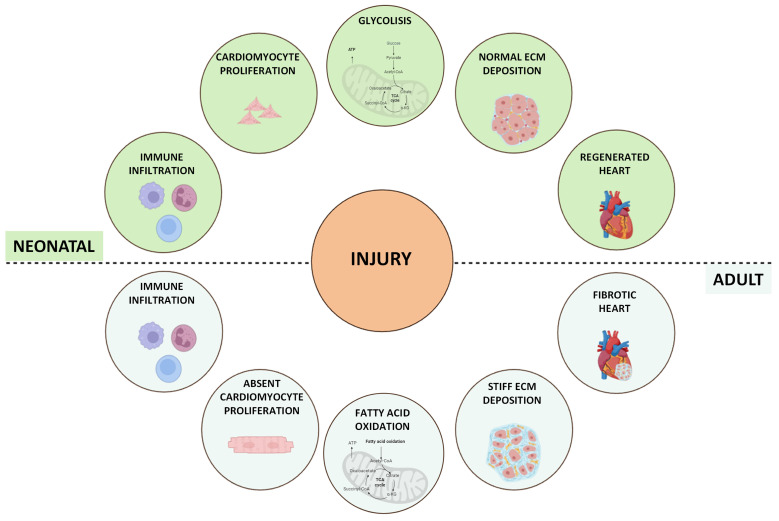
Schematic overview of the response to myocardial injury in neonatal and adult mammalian models. After myocardial injury, different events can occur to regenerate the damaged heart. Neonatal mice (aged < 1 week) are capable of full regeneration and functional recovery after injury. In adult mice and humans, this regenerative capacity is lost, and the necrotic tissue after injury is replaced with a fibrotic scar.

The term “cardiac regeneration” encompasses a broad spectrum of concepts surpassing CM’s simple renewal potential. It involves factors such as growth factors (GFs), paracrine signals, transcription factors, and miRNAs, all of which have demonstrated significant roles in promoting CM proliferation and angiogenesis in animal models. This suggests promising avenues for enhancing the intrinsic regenerative ability of the adult mammalian heart [16]. Among the various models of vertebrates where cardiac regeneration has been extensively researched and whose CMs can proliferate and regenerate injured cardiac tissue, the neonatal mammal stands out [17]. This model, during the early stages of life, displays significant regenerative potential with full regenerative capability following different types of injuries, such as cryoinjury, apical resection, left anterior descending (LAD) coronary artery ligation, pulmonary artery binding (PAB), and transverse aortic constriction (TAC) [18]. However, this regenerative potential is rapidly lost after birth, starts to decline at P3 (3 days old), and becomes negligible at P7 [19]. An in-depth study of the differences between P1 and P3 neonatal hearts reported an increase in ECM stiffness at 3 days, which appeared responsible for the loss of regenerative ability [20] (Figure 1). Recently, an emerging role of metabolic switches in controlling cell functions, such as proliferation, differentiation, and paracrine signals, which are crucial for development and regeneration, as well as in regulating cell fate, has been identified. In neonatal mice, heart energy production primarily involves glucose and lactate oxidation, supported by heightened glycolytic activity to facilitate rapid cardiac growth. Conversely, a significant metabolic transition has been observed in postnatal mice, where CMs predominantly use mitochondrial fatty acid oxidation for energy production to sustain heart maturation and function [21]. It has been demonstrated that mTORC1 inhibition specifically accelerates metabolic maturation post-MI compared to sham controls in neonatal mice, reducing the CM proliferation rate and increasing CM size following MI [22]. The shift from glycolysis to fatty acid oxidation during postnatal heart maturation correlates with the inability of the adult heart to regenerate following injury and may represent a target to promote heart regeneration. In both mouse and human adult hearts, the response to injury results in suboptimal regeneration, leading to pronounced fibrotic scar formation. This causes cardiac remodelling, which includes ventricular wall thinning and dilation, ultimately compromising cardiac contractility [23].

Over the past 15 years, extensive efforts have been made to identify the genes and pathways involved in myocardial regeneration following cardiac damage [17]. The bulk of changes in gene expression within cardiac cells occur during ontogeny, transitioning from the neonatal to the adult phenotype. Contrary to a paradigm where a specific genetic program is exclusively activated upon regenerative demand, neonatal cardiac cells exhibit an inherent predisposition to sustain cell cycle activity from early developmental stages [24]. During neonatal heart development, the percentage of ECs increased in the first days after birth, suggesting EC proliferation and angiogenesis during this period [25]. Furthermore, transcriptomic analyses of both murine neonatal and adult CMs and ECs confirmed a strong difference in transcriptional profiles after MI. While neonatal (P1) CMs and ECs are enriched in cell-cycle-associated transcription factors, adult (P56) CMs and ECs failed to reactivate neonatal transcriptional networks after MI [26].

Studies performed using large animal models seem to corroborate these results. However, the authors highlighted that, similarly to murine models, the CM regenerative capacity was lost shortly after birth in neonatal swine and ovine [27,28,29]. A few data are available on the regenerative capacity of the human heart. Mallova and co-workers reported that CM proliferation contributes to developmental heart growth in young human beings (between 1 and 20 years old) [30]. This endogenous regenerative capacity was also observed in one case report of an infarcted human newborn, where the neonatal heart recovered myocardial structure and function after reperfusion therapy [31].

The growing interest in studying tissue regeneration in the adult heart was encouraged by the documentation of a modest regenerative potential within adult cardiac tissue, challenging the conventional view of the adult heart as a terminally differentiated (post-mitotic) organ [16]. Specifically, it has been demonstrated that CMs exhibit an approximate annual renewal rate of 1% at the age of 20, a rate that gradually diminishes with increasing age, ultimately reaching 0.3% by 75 years [32]. Consequently, less than half of myocardial tissue undergoes regeneration throughout an individual’s lifespan. This observation, coupled with the evidence of robust neonatal cardiac regenerative capacity, has spurred investigations into strategies aimed at enhancing endogenous cardiac regeneration. Furthermore, the presence of concomitant diseases, as frequently observed in elderly patients, can negatively affect the heart’s regenerative capacity [33]. Stem cells are crucial for maintaining tissue homeostasis and facilitating the repair of damaged tissues. Consequently, their dysfunction or depletion with age can significantly impair the tissue’s ability to repair and regenerate [34]. Chronic inflammation, oxidative stress, metabolic dysregulation, and age-related multimorbidity negatively impact the endogenous cardiac stem/precursor cell population, which plays a crucial role in myocardial cell turnover and repair following injury [35]. Ageing inherently leads to alterations in cellular signalling and stem/precursor cell activation to counteract the existing cellular senescence within the tissue. In particular, senescent cells affect regenerative niches via the release of pro-inflammatory secretomes, which hinders the proliferation and regeneration of stem cells [36]. In the context of tissue injury, as occurring in MI, this can impair repair and regeneration [37]. In diabetes, for instance, acute hyperglycaemia induces human cardiac stem cell death by upregulating matrix metalloproteinase-9 (MMP9), which promotes apoptosis and pyroptosis [38]. Additionally, diabetes negatively affects cardiac repair by promoting fibrosis, altering metabolic pathways, and reducing the regenerative response of CMs and ECs [35], thus exacerbating cardiac dysfunction and limiting the potential for recovery following MI [39].

## 2. Myocardial Regeneration

Myocardial regeneration represents a crucial area of research in the field of biomedicine, aimed at replacing damaged CMs, particularly following MI. Despite significant advancements in recent years, myocardial regeneration remains a complex challenge, characterised by scientific debates regarding the identification of cardiac precursors and their actual regenerative potential. In this framework, most of the recent studies discussed in this revision have increasingly focused on investigating the role of the paracrine signalling of stem/precursor cells rather than their direct differentiation during endogenous regeneration [40,41,42]. These studies indicate that modulating paracrine signals may ultimately enhance cardiac regeneration and improve the control of unwanted side effects. Together, stem/precursor cells, GFs, and cell cycle regulators are the main protagonists in stimulating CM proliferation and improving cardiac function; therefore, they might be the key to unlocking effective new therapies for the regeneration of damaged cardiac tissue [43].

### 2.1. Stem/Precursor Cells in Myocardial Regeneration

Stem/precursor cells have the ability to self-renew and differentiate into functionally mature specialised cells in various human tissues [44]. Over the past decade, there have been intense debates regarding the existence of cardiac stem cells and their potential function, particularly on the role of c-Kit-positive stem cells in cardiac regeneration. Kit (CD117) is a type III receptor tyrosine kinase that activates a downstream signalling cascade upon binding to the stem cell factor. Studies conducted in the last 20 years have demonstrated the expression of c-Kit in various cell types [45] and also in a population of stem cells in the adult heart [46]. Subsequent studies aimed at validating or expanding these works have led to significant conflicts and controversies, primarily focused on inconsistent results and discordant conclusions arising from the use of distinct methodologies and models, concluding that cKit+ progenitor cells are not a relevant source of CMs in vivo [47]. These findings raised questions about the interpretation of previous data and suggested that the regenerative capacity of the heart may have been overestimated [48]. Though the research aimed at tracing stem cells using knock-in Cre/Lox and/or Dre/Rox models has been partially criticised [49], as well as the one using inducible transgenic reporters [50], today, the role of stem/precursor cells in myocardial regeneration remains a challenging field of cardiac research that lacks definitive and indisputable scientific evidence. In 2019, Chien and colleagues provided a comprehensive analysis of the debate surrounding the existence of cardiac stem/progenitor cells, arguing that overemphasising these cells as the primary drivers of cardiac regeneration may not be the most productive path forward. Moreover, the publication underscores the need to shift the focus of cardiac cell therapy research toward a better understanding of paracrine mechanisms and the promising potential of iPSCs [47]. However, the heart harbours a diverse population of c-Kit+ cells, primarily consisting of endothelial progenitor cells (EPCs), and, for this reason, it is important to underline that c-Kit expression is necessary but not sufficient for identifying true adult stem/precursor cells [51].

**Figure 2:**
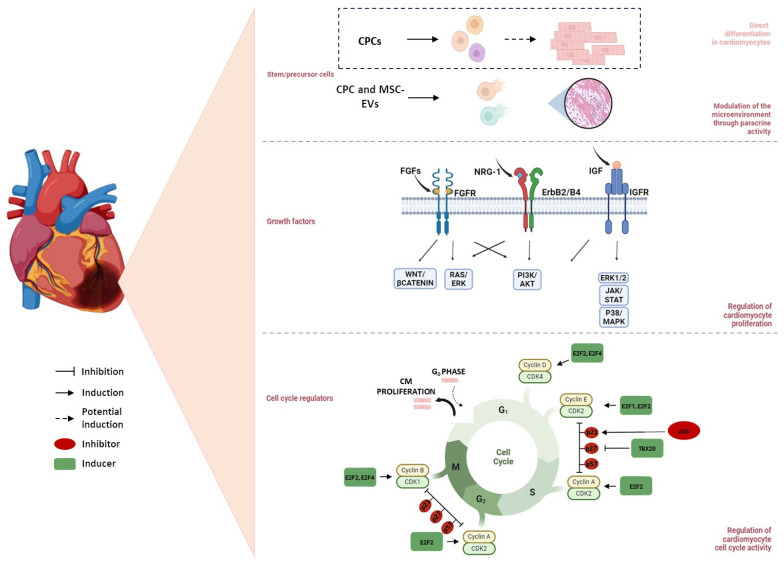
Regulatory mechanisms involved in myocardial regeneration. It is widely debated whether cardiac progenitor cells (CPCs) possess the potential to differentiate into cardiomyocytes (CMs) in the adult heart. Mesenchymal stem cells (MSCs), through the paracrine release of exosomes, modulate the microenvironment, thereby promoting tissue regeneration. Growth factors, such as Fibroblast growth factors (FGFs), Neuregulin-1 (NRG-1), and Insulin-like growth factor (IGF), regulate CM proliferation by activating key molecular pathways, including WNT/β-catenin, PI3K/AKT, and JAK/STAT, which contribute to myocardial regeneration. Additionally, cell cycle regulators, such as cyclins, p53, and transcription factor T-box 20 (TBX20), govern CM cell cycle re-entry, supporting cardiac repair and regeneration.

Nevertheless, the goal of this review is to provide the most significant recent findings about the role of stem cells in cardiac regeneration after MI, particularly concerning cardiac progenitor cells (CPCs) and mesenchymal stem cells (MSCs) (Figure 2).

#### 2.1.1. CPCs

CPCs constitute a heterogeneous population of resident cardiac cells distributed throughout the heart [52]. CPCs are immature yet committed myocardial cells capable of proliferation and differentiation into major cardiac cell types, namely CMs, SMCs, and ECs, thereby possibly facilitating the regeneration of damaged cardiac tissue and promoting neovascularisation [53]. Several types of CPCs have been identified in cardiac development and regeneration at various stages that can be classified based on the expression of different surface markers (c-Kit, Sca-1, Mesp1, KDR/Flk-1) and their locations within the heart (mesoderm, epicardium, side population, or cardiosphere-derived) [54].

##### c-Kit+ CSC/CPCs

c-Kit+ CPCs contribute to new CM generation during embryonic development and the early postnatal period. This capacity decreases in the adult heart, with only a few new CMs originating from CPC [55]. Vicinanza et al. demonstrated the presence of small niches of c-Kit+ resident CSCs that constitute less than 1% of the c-Kit+ cardiac cell population that possesses clonogenic potential. The entire population of c-Kit+ cardiac cells was compared with clonogenic c-Kit+ CSCs in a murine model of MI to demonstrate the differences in their regenerative capacities. Following the induction of MI in rats, both the total c-Kit+ cardiac cells and the clonogenic c-Kit+ CSCs were injected directly into the myocardium near the infarct border zone. Twenty-eight days post-MI, the clonogenic c-Kit+ CSCs persisted in the infarcted hearts and contributed to robust myocardial regeneration. These CSCs generated new mononucleated CMs, arterioles, and capillaries. Moreover, the clonogenic CSCs significantly reduced myocyte apoptosis, hypertrophy, scar size, and left ventricular dilation, leading to a marked improvement in cardiac function compared to control rats, all of which developed HF. Conversely, total c-Kit+ cardiac cells, corresponding to approximately 99% of the c-Kit+ population, exhibited minimal engraftment, primarily confined to the infarct border zone. These cells failed to generate significant numbers of new CMs, while most differentiated into ECs. These findings demonstrate that only a small number of c-Kit+ CSCs possess clonogenic potential, self-renewal capabilities, and multilineage cardiac differentiation potential in vivo compared to the majority of c-Kit+ cardiac cells that exhibit primarily vasculogenic potential [56]. The intrinsic regenerative capacity of CPCs can be enhanced through the use of GFs such as Hepatocyte growth factor (HGF) or Insulin-like growth factor (IGF). It has been demonstrated that a population of resident c-Kit+ CPCs in the adult swine myocardium is activated in response to the intracoronary administration of IGF-1/HGF after MI. This activation promotes myocardial regeneration and microvasculature with a dose-dependent effect. This process results in a significant improvement in cardiac function, a reduction in cell mortality, and an increase in left ventricular ejection capacity. Additionally, the use of small amounts of IGF-1/HGF directly into the coronary artery feeding the infarcted area has been shown to improve CM survival and promote cardiac remodelling, leading to an overall structural improvement in the myocardium observable even two months after the infarction event [57].

##### Sca-1+ CPCs

Sca-1+ CPCs exhibit a mesenchymal phenotype, possess limited potential for cardiogenic differentiation, and can enhance cardiac remodelling following MI [12]. Within the Sca-1+ CPCs, a subpopulation expressing high levels of Bmi1 (Sca-1+ Bmi1+) has been identified, which appears to play a particularly important role in regenerative mechanisms [58]. At first, it has been demonstrated that Sca-1+ Bmi1+ CPCs significantly increase in the peri-infarct area of mice and can contribute to cardiac repair via the de novo generation of CMs after MI, with new CM formation rates reaching 13.8% compared to 4.7% in non-infarcted hearts [59]. Subsequent in vitro investigation has demonstrated that Bmi1+ cells can give rise to both CM-like and smooth muscle-like cells [60]. However, when transplanted into the infarcted hearts of mice, they do not differentiate into new CMs [61]. More recently, studies indicate that Sca-1+ Bmi1+ CPCs are located around the vascular structure, exhibit an endothelial-related phenotype, and contribute to neovascularisation after MI [62,63,64,65]. Indeed, the ablation of Sca-1+ Bmi1+ CPCs impaired the angiogenic response following MI [66] and, with age, oxidative stress confines Bmi1+ progenitor cells to perivascular regions of the heart, reducing their ability to respond to damage [67].

Thus, the positive effects of Sca-1+ cell transplantation are likely due to their role in promoting angiogenesis and exerting paracrine effects rather than direct CM differentiation.

##### Mesp-1+ CPCs

Cells expressing Mesp-1 mainly contribute to the formation and early elongation of the heart tube during development [68]. Mesp1-CPCs differentiated into cardiac myocytes, vascular SMCs, and ECs in a mouse model of MI. In particular, mice injected with CPCs after MI exhibited significant improvements in cardiac pump function and overall survival rates. The differentiation of cardiac myocytes was primarily confined to the infarct and border zones and occurred less frequently than the differentiation into the other two lineages. CPC-derived vascular SMCs and ECs were abundant in both the infarct and border zones, often contributing to neovasculogenesis and showing co-staining with proliferation markers [69].

##### KDR/Flk-1+ CPCs

The kinase insert domain receptor (KDR), also known as Flk-1 or vascular endothelial growth factor receptor 2 (VEGFR2), is a progenitor marker present in the early stages of cardiac development in humans [70]. KDR/FLK1 is frequently used in combination with other cardiac markers, such as platelet-derived growth factor-alpha (PDGFRα), C-X-C chemokine receptor type 4 (CXCR4), and, sometimes, Mesp-1 [71]. High KDR/Flk-1 expression correlates with differentiation towards haematopoietic lineages. CPCs with KDR/Flk-1 low expression stimulate cells to follow cardiac differentiation and can generate a second population of FLK1+, which represents the first multipotent cardiac progenitor cells permanently committed to the cardiogenic fate [72,73].

##### CPC Location Within the Heart

CPCs have been found in the mesoderm’s first and second heart fields (FHF and SHF) and can be isolated from cardiospheres obtained from cardiac biopsy, as well as from the side population (SP-CPCs) and epicardium (EPDCs). These CPCs share the ability to contribute to cardiac development but differ in specific markers, differentiation potency, and regenerative capacity in the adult heart. FHF- and SHF-derived CPCs are crucial for embryonic development, with SHF CPCs exhibiting greater differentiation potential [54]. SP-CPCs, EPDCs, and cardiosphere-derived CPCs demonstrate putative differentiation potential, although their key role in tissue repair and modulation of the local environment is attributed to their robust paracrine activity facilitated through the secretion of biologically active extracellular vesicles (EVs) [74]. EVs represent a diverse population of membrane-enclosed vesicles released by normal, apoptotic, and tumour cells, notably categorised into different types: exosomes, ectosomes, apoptotic bodies, and oncosomes [75]. Given the significant paracrine impact of stem cells in tissue repair, including cardiac tissue regeneration, extensive research has been conducted to harness stem-cell-derived EVs, particularly exosomes, as promising next-generation therapeutic agents in cardiac repair [76]. The epicardium plays a fundamental role in cardiac development, contributing both cellular elements and paracrine factors essential for heart formation. These epicardial processes are not only vital during development but are also recapitulated in the adult heart following cardiac injury, albeit less efficiently [77]. The epicardium may therefore function as a reservoir of progenitor cells [78], which can release EVs that enhance the proliferation of neonatal murine CMs in vivo. When injected into the injured area of infarcted hearts, EPDC-derived EVs promoted CM cell cycle re-entry, doubling the number of proliferative CMs compared to the control group. The induction of CM proliferation by EPDC-derived EVs was observed in both P1 and P7 mouse hearts. This proliferation was induced by microRNAs found in the EVs that lead to the activation of the Akt, Hippo, and ERK signalling pathways [79].

#### 2.1.2. MSCs

On the other hand, MSCs represent the most undifferentiated stem cells involved in tissue repair across various organs. As stromal cells, they possess a unique capacity for self-renewal and exhibit multilineage differentiation potential [80]. Although there is no concrete evidence regarding their direct intervention in myocardial regeneration through transdifferentiation, researchers have increasingly become interested in MSCs in recent years due to their robust regenerative capabilities and their greater accessibility, especially in the investigation of ischaemic heart disease treatments [81,82]. MSCs secrete a range of bioactive molecules, including GFs, cytokines, chemokines, and EVs, which can modulate the cardiac microenvironment and promote tissue repair processes [83,84,85]. Currently, investigation of MSC-Exosomes (MSC-Exo) and MSC immunomodulatory properties are major research focuses.

##### MSC-Exosomes

In the past few years, studies have provided evidence that MSCs function as regulators through the secretion of exosomes transporting genetic materials (microRNA, messenger RNA) and proteins (tetraspanins, annexins, heat shock proteins) to target cells [76,86,87,88]. Considering its effect on the regulation of cell death and proliferation, miR-199a-3p was implicated in stimulating CM proliferation [89], and it was found abundant in MSC-Exo [90]. miR-199a-3p has been determined to be one of the few miRNAs crucial for the induction of cardiac regeneration. Targeting Crim1, a gene essential to CM proliferation inhibition, miR-199a has been found to increase in vitro CM proliferation. Infarcted mouse hearts were injected with adenovirus vectors expressing miR-199a-3p, and, after 60 days, Edu-positive CMs were detected in the infarct border zone, confirming that miR-199a-3p stimulated their proliferation [91]. However, the persistent and uncontrolled expression of this microRNA was associated with sudden arrhythmic death in a swine model, suggesting that, in large mammals, such therapies need to be tightly dosed [92]. MSC-Exo may also overexpress miR-210, which protects myocytes from in vitro and in vivo stress [93]. miR-210 promoted cardiac regeneration, inducing CM proliferation in adult mice post-MI with a significant increase in either Edu, Troponin T, or Aurora B positive CM in the peri-infarct area. In addition, mice treated with miR-210 showed a significant upregulation in their β-catenin level, supporting CM cell cycle progression [94].

##### MSC Immunomodulatory Properties

MSCs possess immunomodulatory properties that can regulate the immune response and create a favourable environment for cardiac repair. It has been demonstrated that MSCs suppress the activation and proliferation of immune cells, such as T cells, B cells, and natural killer cells, and inhibit the production of pro-inflammatory cytokines [95]. This immunomodulatory effect can attenuate excessive inflammation, which is harmful to cardiac tissue, and promote a more balanced immune response that favours tissue repair and regeneration [96]. Accumulating evidence indicates that MSCs contribute to cardiac repair through modulating macrophage functions. The recent findings on MSC-Exo highlighted that miR-182, contained in MSC-Exo, can preserve heart function in I/R injury mice by inhibiting the expression of TLR4 and enhancing M2 polarisation [97]. Moreover, miR-21-5p plays a role in cardiac repair by inducing macrophage polarisation towards the M2 phenotype, which in turn reduces the inflammatory response following injury [98].

### 2.2. Growth Factors

GFs are molecules capable of stimulating several cellular processes, including cell proliferation, migration, differentiation, and multicellular morphogenesis, during development and tissue healing [99]. Given their direct impact on these cellular functions, GFs have been extensively investigated for their role in regulating CM proliferation [100]. GFs may induce stem cell recruitment, anti-apoptotic and/or angiogenic effects, adult CM proliferation, and ECM remodelling. All these mechanisms can contribute to promoting tissue regeneration and improving cardiac function, inducing CM proliferation in pathological conditions such as infarction-induced damage (Figure 2).

#### 2.2.1. Neuregulin-1

Neuregulin-1 (NRG-1) is a growth factor involved in various processes of cardiac development, including CM proliferation and differentiation. Primarily produced and released by vascular ECs, it acts through its binding with the ErbB4 receptor [101]. This binding induces ErbB2-ErbB4 heterodimerization, and the resulting intracellular signal is capable of activating the Ras/ERK, PI3K/Akt, and Src/Fak pathways, which regulate multiple functions of CMs such as growth, proliferation, survival, and structural sarcomeric organisation [102]. The regulatory role of NRG-1 in cardiac development is predominant during the neonatal phase compared to adulthood in mammals, primarily due to the decrease in the cardiac level of ErbB2, which is essential for heterodimerization with ErbB4 [103]. This reduction of ErbB2 was observed in the heart at P7 and further decreased at P28, resulting in the loss of cardiac regenerative capacity. These findings on the role of NRG-1 in cardiac regeneration suggest that combinatorial overexpression strategies of NRG-1 and ErbB2 might be a therapeutic target to initiate cardiac repair after MI.

In mammals, a metabolic switch from glucose to fatty acid metabolism occurs in the first postnatal week and coincides with increased mitochondrial activity and loss of regenerative potential [104]. Although constitutive expression of ErbB2 in the in vitro experiments led to a metabolic shift toward glycolysis, which is beneficial in promoting CM cell cycle re-entry, in the in vivo adult murine model, its constitutive expression induced hypertrophic cardiomyopathy [105,106]. This research group also developed a transgenic mouse model in which the expression of ErbB2 could be transiently induced with Doxycycline. They used a young mouse model (P8) already exited from the regenerative window (MI induced at P7) and a 5-week adult mouse model (MI induced at P42), and subsequently administered Doxycycline for 10 and 21 days, respectively, to achieve transient expression of ErbB2 signalling. This transient expression resulted in a significant improvement in cardiac recovery after MI, with an increase in proliferating CMs (TnT+, Ki67+, and Aurora B+) together with a reduction in scar size in both young and adult models compared to controls [103]. This improvement was particularly pronounced after the cessation of ErbB2 expression, especially in the adult model. Analyses of sarcomeric structures conducted one month after infarction (approximately P70) and ErbB2 induction revealed the presence of robust dedifferentiated cardiac muscle. Moreover, one month after the cessation of ErbB2 expression (approximately P98), both tissue morphology and sarcomeric organisation were restored, indicating the process of CM redifferentiation leading to reduced scar formation and improved cardiac function after MI. These results suggested that a transient expression of ErbB2 in the adult model is sufficient to reopen the regenerative window of CM proliferation. In addition, the analysis of downstream mediators of ErbB2 proved these effects in CMs to be mediated by ERK, AKT, and GSK3β/β-catenin pathway activation [106]. Recent studies have highlighted that in the process of cardiac regeneration mediated by ErbB2 in an adult murine model of MI, an epithelial-mesenchymal transition (EMT)-like process is also involved; thus, the authors suggested that CM migration is an essential element for cardiac repair and scar replacement by new CMs, together with ECM and cytoskeletal remodelling. Furthermore, these authors pointed out the role of YAP, a downstream mediator of the ErbB2 and ERK signalling pathways, the phosphorylation of which is necessary to induce cytoskeletal remodelling and regulate CM proliferation [107]. CM dedifferentiation is associated with an upregulation of foetal gene expression, the disassembly of the sarcomere, and changes in cellular morphology, playing a key role in tissue regeneration [108]. The activation of the EMT process is therefore crucial in promoting the reactivation of efficient regenerative processes in tissues that have lost this capability, such as cardiac tissue. In particular, the transient expression of the ErbB2-YAP signalling axis can lead to the activation of EMT, causing dedifferentiation of adult CMs and their subsequent redifferentiation when ErbB2-YAP expression is stopped. However, only a transient activation is required, while a constitutive activation of ErbB2 can lead to cardiac hypertrophy. This phenomenon has also been confirmed in various murine models following MI [109].

#### 2.2.2. Fibroblast Growth Factors

Fibroblast growth factors (FGFs), comprising a family of 22 members in mammals, play a crucial role in cardiac function, spanning from developmental processes to maintaining homeostasis and contributing to disease progression [110]. Canonical FGFs, such as FGF1-10, FGF16-18, FGF20, and FGF22, are small proteins secreted in the ECM by various cell types, including ECs, FBs, CMs, and MSCs, and function either as paracrine or autocrine GFs [111]. Hormone-like FGFs, such as FGF15, FGF19, FGF21, and FGF23, are released into the blood and regulate different aspects of metabolism. Additionally, there is a subfamily of FGFs comprising four intracellular proteins, namely FGF11-14, which play a role in modulating ion channels [111]. Notably, FGF 1, 2, and 10 mediate a crucial role in the heart by binding to their receptors (FGFRs) [112,113]. The potential of FGF1 has been further underscored through its synergy with NRG-1, an agonist of the epidermal growth factor receptor tyrosine kinase, in both rat and swine models. Both GFs were administered directly into the myocardium, encapsulated within microparticles, four days post-MI. The combination of these growth factors in infarcted hearts led to a significant improvement in cardiac function, with a reduction in necrotic area and appropriate remodelling of the heart compared to the untreated control. Interestingly, a decrease in the number of apoptotic CMs was observed after 3 months of treatment, with an increase in proliferating CMs (cardiac TnT+ Ki67+) in the infarcted zone following treatment [114].

The role of FGF10 in adults is involved in maintaining proper cardiac morphology and, particularly, in preserving the correct thickness of the ventricular wall through the regulation of CM proliferation. Notably, FGF10 controls the CM cell cycle by binding to FGFR2b during embryonic development, while in the adult heart, it exerts this control by binding to FGFR1b [115]. The expression of FGF10 changes during the developmental stages: gene expression analysis in both foetal and postnatal/adult hearts showed that FGF10 expression decreased from 18.5 days of foetal growth (E 18.5) until P10, when CMs exit from the cell cycle. FGF10 expression then increased again at P16, reaching maximal levels at P56 (8 weeks old) before decreasing again at P100 (14 weeks old). These results support a physiologically relevant role for FGF10 in controlling CM proliferation both during foetal development and in the postnatal/adult heart [110,116]. Deletion of FGF10 compromises CM proliferation, as evidenced by the significant reduction in Ki67+ CMs in the right ventricle of FGF10^−/−^ embryos [117]. During foetal development, FGF10 action involves the phosphorylation of FOXO3 and downregulation of the cyclin inhibitor p27. In the adult transgenic mice model, the analyses of the percentage of CM positive for the proliferative marker Ki67+ and mitotic marker PH3+ (phosphorylated histone 3) reveal that FGF10 overexpression (for 14 days) specifically promotes CM re-entry into the cell cycle. These authors attributed the cell proliferation exclusively to CMs rather than non-myocytic cells, as evidenced by the presence of α-actinin staining. In particular, these smaller and mononucleated new CMs were observed in the infarcted and border zones [117]. The role of FGF10 in cardiac regeneration has also been confirmed using mice with reduced FGF10 expression (FGF10^+/−^). Twenty-one days post-MI, endogenous levels of FGF10 increased in CMs, and these FGF10^+/−^ mice exhibited worsened cardiac performance, including further decreases in ejection fraction and fractional shortening and further increases in left ventricular volume compared to WT. Interestingly, decreased FGF10 levels compromised CM proliferation and impaired fibrosis post-MI. Immunofluorescence staining for Ki67, PH3, and Aurora B revealed significant impairment in CM proliferation in FGF10^+/−^ mice compared to WT, both at 5 and 21 days after MI. At the same time points, histological analysis with Sirius Red staining showed an increase in fibrotic tissue together with upregulated collagen gene expression in FGF10^+/−^ mice [117]. These results reinforced the concept that high levels of FGF10 are necessary for cardiac regeneration and the preservation of cardiac function, preventing fibrosis, and reducing post-infarction cardiac remodelling by modulating the gene expression of key regenerative signalling pathways, including the transcription factor Meis1, which controls CMs’ and ECs’ fate, the Hippo signalling pathway, and a pro-glycolytic metabolic switch. Most importantly, in the same study, the pivotal role of FGF10 in cardiac regeneration has also been confirmed in failing human heart biopsies, where FGF10 expression significantly correlated with smaller CM cross-sections and enhanced Ki67+ CM numbers in the border zone, thus reinforcing the conclusion that FGF10 promoted CM renewal [117].

#### 2.2.3. Insulin-like Growth Factors

IGF1 and IGF2 are growth factors that play a central role in activating endocrine, paracrine, and autocrine signalling pathways in cardiac development. While IGF1 is predominantly present in the postnatal period, including adulthood, IGF2 prevails during embryonic development [118].

Indeed, IGF-1 receptor inhibition has been shown to hinder CM proliferation, thereby impeding cardiac development and regeneration in the mouse heart [119]. The main action of IGF1 involves the phosphorylation of MEF2C, resulting in p38-MAPK pathway activation. Additionally, it activates several other signalling pathways, including ERK1/2, PI3K, PKC, PKB, Jak/STAT, and PLC [120].

Overexpression of IGF2 in parthenogenetic stem cells (PSCs) accelerates their differentiation into CMs, resulting in a promising strategy for enhancing cardiac regeneration [121]. Shen et al. recently reported that IGF-2 invalidated the intrinsic regenerative effects in P1-day mice, highlighting the critical role of IGF-2 as a mitogen in neonatal mice [41].

The bioavailability of IGF is regulated by six members of the IGF-binding protein (IGFBP) family [122].

IGFBP1 regulates cell proliferation, migration, and metabolism by activating integrin-ILK/FAK/PTEN signalling through integrin receptors on the cell membrane [123]. Under hypoxic conditions, IGFBP1 influences myocardial cell apoptosis by regulating Hypoxia-Inducible Factor(HIF)-1α. Its role as a negative regulator of CM proliferation has also been demonstrated in an MI mouse model. Seven days after intramyocardial injection of adenovirus-shRNA-IGFBP1, the animal hearts were subjected to LAD ligation. Silencing IGFBP-1 can reduce infarct size and attenuate apoptosis in CMs, with decreased interstitial fibrosis in the infarcted zone of the shRNA-IGFBP1 group compared to controls. Additionally, immunofluorescence staining showed an increased number of Ki67+ and pH3+ CMs in the border zone of the shRNA-IGFBP1 group compared to the control group, suggesting that knocking down IGFBP-1 promotes cardiac regeneration and reduces fibrosis after MI [124].

Wang et al. identified a regulator of myocardial cell proliferation in the neonatal heart, IGF2BP3 [24]. IGF2BP3 expression is enriched in the regenerative heart following MI and promotes CM division, resulting in a potential target for future cardiac regeneration therapy [24]. IGF2BP3 promoted myocardial regeneration in adult mice by stabilising MMP3 mRNA through the interaction with m6A (N6-methyladenosine) modification. In particular, adeno-associated virus 9 (AAV9)-packaged IGF2BP3 was injected into the myocardium of the left ventricle of P1 mice immediately after MI induction. At 7 and 56 days after IGF2BP3 injection, CM proliferation was markedly increased in P7 and adult mice compared with groups injected with the control vector, as determined by immunostaining of pH3 and Ki67. Furthermore, overexpression of the IGF2BP3 significantly increased the proportion of proliferative CMs in the marginal zone of the MI heart at 14 days [125]. IGF2BP3, along with the cytokine Ccl24, is highly expressed in macrophages of P1 neonatal mice compared to P14. This expression is thought to support cardiac regeneration by promoting postnatal CM proliferation [126].

Taken together, these results highlight the role of IGFs in myocardial regeneration, supporting CM proliferation. Further investigation into their involvement in cardiac repair processes and immune cell regulation could offer new insights for enhancing myocardial regeneration after injury.

### 2.3. Cell Cycle Regulators

#### 2.3.1. Cyclins

Various regulators of cell cycle checkpoints, including cyclins, cyclin-dependent protein kinases (CDKs), CDK-activating kinases (CAKs), and their inhibitors (CKIs), have been identified as key players in regulating the cell cycle activity of CMs during both prenatal and postnatal development [127].

CDKs are activated by complex formation with cyclins, leading to advances in the cell cycle. In mammals, the complexes are Cyclin D-CDK4/6 (G1 phase), Cyclin E-CDK2 (G1/S phase), Cyclin A-CDK2/1 (S/G2 phase), and Cyclin B-CDK1 (M phase) [128]. CDK activities are regulated through interactions with CKIs such as p21, p27, and p57, which are implicated in CM cell cycle arrest. It has been demonstrated that the simultaneous knockdown of p21, p27, and p57 with siRNA induces both neonatal and adult CMs to enter the S-phase and undergo mitosis in vitro [129,130,131]. When complexed with CDK4 or CDK6, D-type Cyclins drive cell cycle re-entry from G0 to G1 phase. Protein levels of D-type Cyclins and activating kinases (i.e., CDK4 and CDK6) have been observed to sharply decline in the early postnatal stages and adulthood [112]. In particular, Cyclin D1 is expressed at very low levels in adult CMs, allowing the maintenance of a quiescent state of these cells. Indeed, specific induction of Cyclin D1 expression in adult mouse CMs leads to an increase in proliferation markers, such as BrdU, Ki-67, and PCNA (Proliferating cell nuclear antigen), as well as an increase in the expression of DNA replication-related proteins, resulting in more than 40% of CMs re-entering in the cell cycle [132]. Recently, it has been demonstrated that miR-301a, enriched in neonatal CMs, is able to induce cell cycle re-entry and enhance cellular proliferation in H9C2 cells and primary CMs by upregulating Cyclin D1. In vitro assays (Ki67, EdU, Aurora B stainings), confirmed that miR-301a overexpression led to increased proliferation and G1/S transition of CMs. AAV9-mediated cardiac delivery of miR-301a promoted cardiac repair and regeneration in an MI murine model, via an increased expression of Cyclin D1 by downregulating PTEN and upregulating phosphorylated AKT and GSK-3β. In the miR-301a-treated mice, 1.5–2.0% of adult CMs were double positive for α-actinin and Ki67, compared to 0.5–1.0% in the control mice, indicating the improved proliferative ability of CMs by miR-301a in vivo [133].

Cyclin B1 interacts with Cyclin-dependent kinase 1 (CDK1) to orchestrate the transition from the G2 phase to mitosis. This complex starts to increase its activity during late G2, through prometaphase until the cell enters metaphase [134]. Scientific evidence in both in vitro and in vivo models demonstrated the importance of both Cyclin D1/CDK4 and Cyclin B1/CDK1 complexes in the cell cycle re-entry of CMs. At first, Tamer et al. proved that a cocktail of Cyclin D1/CDK4 and Cyclin B1/CDK1 efficiently enhances the proliferation of hiPS-CMs, primary mouse neonatal (isolated at P7) and adult rat (isolated at 4 months) CMs. Subsequently, the authors validated these results in an in vivo adult mouse model of coronary ligation with intramyocardial injections of adenoviral vector encoding for CDK1, Cyclin B1, CDK4, and Cyclin D1, showing that adult CMs expressing these four factors underwent stable cell division, resulting in a significant improvement in cardiac function after acute or subacute MI. Interestingly, a similar improvement was obtained even with injection after the formation of scar tissue. Moreover, it has been noted that the use of inhibitors of TGFβ results in an unblocking of adult CM replicative ability by promoting the function of G1 phase cyclins, likely attributable to an indirect suppression of the CDK inhibitor p27 [135].

Cyclin D2 is well known as a key regulator of the cell cycle, particularly in the G1 phase, critical for DNA synthesis and cell proliferation. Specific expression of Cyclin D2 in CMs has a significant impact on their proliferative capacity and cardiac regeneration after MI. Indeed, cardiac-specific expression of Cyclin D2 in both adult mice and pigs leads to increased DNA synthesis up to 150 days post-infarction, increasing CM proliferation and regression of the infarct area. In particular, 7 days after MI induction and treatment, immunofluorescence analysis pointed out a significantly higher CM number expressing Ki67, PH3, and Aurora kinase B in the border zone of mouse hearts treated with CyclinD2-modified mRNA compared to control groups. Similarly, a 5.99-fold increase in Ki67+ and a 4.17-fold increase in PH3+ CM were observed in the border zones of infarcted pig hearts 3 days following intramyocardial administration of Cyclin D2-modified mRNA, compared to control groups [136]. D-Cyclin activity can be modulated by dual-specificity tyrosine-phosphorylation-regulated kinase 1A (Dyrk1a), which plays a critical role in controlling the progression of the CM cell cycle. Specifically, inhibition of Dyrk1a led to increased expression of D-Cyclins, promoting the activation of the Rb/E2F signalling pathway. The E2F transcription factor family is crucial for CM proliferation. E2F1, 2, and 3 regulate gene expression that influences entry into the cell cycle, especially the G1/S transition. E2F activity is modulated by various proteins, including the retinoblastoma family protein (pRB) [137,138]. Consistent with these findings, DYRK1a can enhance CMcycling and improve heart function in a mouse model of MI. Specifically, when DYRK1a was ablated in mice, there was an upregulation of cell cycle genes and a downregulation of genes related to contractile proteins, compared to the control group [139].

Recently, the role of Cyclin L1 (CCNL1) in CM proliferation has been studied. Gong et al. demonstrated that the presence of CCNL1 increases in the mouse cardiac tissue undergone MI, suggesting a potential interference in the CM proliferation capacity. Gene silencing in murine models confirmed that the absence of CCNL1 promoted CM proliferation and cardiac repair after MI. The negative role of CCNL1 in regulating the CM cell cycle and in post-infarction cardiac repair could probably be attributed to its binding with CDK11 [140,141].

In addition to the cyclins mentioned above, Cyclin A2 also plays a role in the regulation of the cell cycle. It regulates two different stages of the cell cycle; specifically, it causes the entry in the S phase when it combines with CDK2, while it allows the cell to progress to phase M when it binds with CDK1. Indeed, the almost total absence of Cyclin A2 in the adult heart has been correlated with the exit of CMs from the cell cycle in mammals [112]. Several scientific studies in different animal models demonstrated the crucial role of Cyclin A2 in CM cell cycle regulation after cardiac injury. In cardiac tissue, elevated levels of Cyclin A2 are crucial in promoting CM cell cycle re-entry two weeks after acute MI in a rat model [142]. Interestingly, the role of Cyclin A2 in post-MI cardiac regeneration has also been confirmed in a porcine heart model [143]. Researchers induced overexpression with intramyocardial injection of replication-deficient adenoviral vectors containing murine Cyclin A2 one week after MI, observing a significant increase in CM proliferation with improvement in cardiac function compared to control after 6 weeks [143]. Notably, other authors pointed out that the Y-box 1 binding protein (YBX1) can stimulate Cyclin A2 activity. Through the use of a circRNA capable of blocking Nfix (Nuclear factor I X), responsible for the degradation of YBX1, it has been possible to restore the ability of YBX1 to bind and activate the Cyclin A2 promoter, thus inducing CM proliferation and inhibiting apoptosis after MI in a mouse model [144] (Figure 2).

#### 2.3.2. p53

The tumour suppressor protein p53 plays a crucial role in regulating both embryonic and postnatal cardiac development. Under physiological conditions, p53 is functionally active in maintaining cardiac structure and regulating the expression of transcriptomes related to metabolism, mitochondrial biogenesis, cardiac architecture and excitation-contraction coupling. It has been demonstrated that the deletion of p53 triggered murine heart hypertrophy and decreased heart function [145,146].

Qi Xiao et al. developed a tracking system for p53+ CMs in mice, covering various stages of development, from neonatal to adult. Using this system, they observed how p53+ CMs responded to myocardial cryo-injury and contributed to cardiac regeneration during the postnatal age [147]. Conversely, elevated levels of p53 in pathological cardiovascular conditions can lead to the activation of various mechanisms, including CMapoptosis, cell cycle arrest, metabolism alteration, and autophagy [148]. These data have been confirmed by Stanley-Hasnain et al., who demonstrated that simultaneous deletion of both p53 and its inhibitor murine double minute 2 (Mdm2) in the adult mouse heart induces CM cell cycle re-entry through the downregulation of Cdk inhibitors p21 and p27 along with Cyclin E-mediated Cdk2 activation. These results can be explained by the ability of p53 and Mdm2 to regulate the G1-Cyclin-CDK complex, which is responsible for maintaining the quiescence of adult mammalian CMs [149]. p53 is also involved in activating autophagy and inducing CM cell death after ischaemic injury. Its inhibition with long noncoding RNAs (lncRNAs) significantly attenuates the activation of cardiac autophagic flux in the ischaemic heart, resulting in a limitation of myocardial infarct size in an adult murine model [150].

The role of p53 in cardiac regeneration can vary significantly depending on the developmental stage under consideration. Variations in the proliferative capacity of CMs and cellular response to cardiac injury can primarily explain the differences in the p53 role between the neonatal and adult periods.

#### 2.3.3. TBX20

During embryonic development, TBX20 plays a pivotal role in driving CMproliferation by binding to the promoters of key genes such as ccna2, cdc6, Mycn, and ErbB2, thereby triggering their transcription [151,152]. Recent research focused on understanding TBX20’s contribution to cardiac regeneration, in particular its ability to maintain foetal characteristics in the adult heart and thereby stimulate CM proliferation. These investigations highlighted the critical role of TBX20 in cardiac regeneration, suggesting that its overexpression could serve as a promising therapeutic approach for cardiac repair after MI. Chakraborty et al. examined TBX20 functions throughout embryonic and foetal development in a murine model overexpressing this transcription factor [153]. They demonstrated its capacity to drive CM proliferation with the consequent thickening of the ventricle wall. The foetal hearts of transgenic mice overexpressing TBX20 exhibited a significant increase in the number of pHH3+ and MF20+ (myosin 4) CMs compared to the control group [153]. The sustained proliferation of CMs induced by TBX20 was linked to increased N-myc1 transcription, a downstream regulator of the TBX20 pathway, alongside the upregulation of connexin-40 and -43 expression throughout the ventricular wall, without altering the signal propagation [153]. In a subsequent investigation, the same research group confirmed an augmented population of small, mononucleated, proliferating CMs and ventricular wall thickening also in the adult transgenic mice model overexpressing TBX20. Moreover, the authors showed that TBX20 preserved foetal characteristics through the activation of Bmp2/pSmad1/5/8 and PI3K/AKT/GSK3β/β-catenin pathways in adult hearts [154]. The impact of TBX20 was further elucidated in a murine model of MI, where its overexpression in adult hearts enabled CM proliferation without compromising cardiac morphology or function [155]. TBX20 overexpression post-MI led to reduced scar size, diminished cardiac hypertrophy, and increased capillary density in mouse hearts, thus preserving cardiac function and enhancing survival of mice four weeks post-MI [155]. Isolated CMs from TBX20-overexpressing hearts showed, besides a recovery of foetal contractile proteins such as MHC and ssTnI (slow skeletal troponin I), a heightened proliferative activity characterised by increased expression of Cyclin A2 and D1 along with reduced expression of cell cycle inhibitors such as p16, p21, and p27 [155]. Genetic silencing of TBX20 in embryonic murine CMs underscored its importance, resulting in a noticeable reduction in the volume of developing ventricular and atrial chambers along with a decrease in CM proliferation due to arrest in the G1-S phase. TBX20 directly represses the genetic programs of cardiac progenitors in adult CMs [156].

In vitro experiments, human-induced CMs (hiCMs) revealed that TBX20, in conjunction with the MGT133 reprogramming cocktail (MEF2C, GATA4, TBX5, and miR-133), induced cardiac reprogramming and activated genes associated with CM contractility and maturation. Notably, CMs induced with MGT+TBX20 exhibited increased beating frequency and enhanced energy metabolism [157].

## 3. Coronary Vascular Regeneration

Following MI, the coronary circulation suffers significant damage due to injury. Initially, there is an increase in vascular permeability as a result of the inflammatory process, leading to oedema. Additionally, platelet, leukocyte, and erythrocyte aggregates can occlude capillary lumens. Furthermore, heightened sympathetic activity in response to injury causes increased constriction of the coronary microcirculatory vessels. All together, these factors can lead to capillary destruction and intramyocardial haemorrhage, exacerbating endothelial and tissue damage and the subsequent inflammatory response [158]. Prolonged ischaemia will inevitably kill all CMs and ECs in the infarct core. ECs make up the largest portion of non-CMs, accounting for about 60% in the adult mouse heart [159]. Starting approximately from the 3rd day post-MI, ECs also contribute to reducing the inflammatory response. Transcriptomic analysis of cardiac ECs from mouse hearts on the 4th day post-MI revealed a significant enrichment of ECs capable of secreting IFN and reducing the inflammatory response by acting on immunosuppressive signalling pathways through PD-L1 and CD39/CD73 pathways [160]. This suggests a potentially significant role of ECs in mitigating the inflammatory response and facilitating the onset of the regenerative response. Indeed, ECs play a crucial role in cardiac regeneration, driving the neovascularisation of the damaged tissue [160,161]. Moreover, the inflammatory environment was able to activate ECs and promote their endothelial-to-mesenchymal transition, and, in particular in the healing process post-MI, this mesenchymal activation favoured EC migration that fostered de-novo vascular network formation [162]. The improvement of circulation in the infarcted area is essential to reduce CM death, especially at the level of the border zone of the infarction, allowing a limitation of the extent of myocardial damage. Vascular networks can be reconstructed through three main mechanisms: angiogenesis, arteriogenesis, and vasculogenesis [163,164].

**Figure 3:**
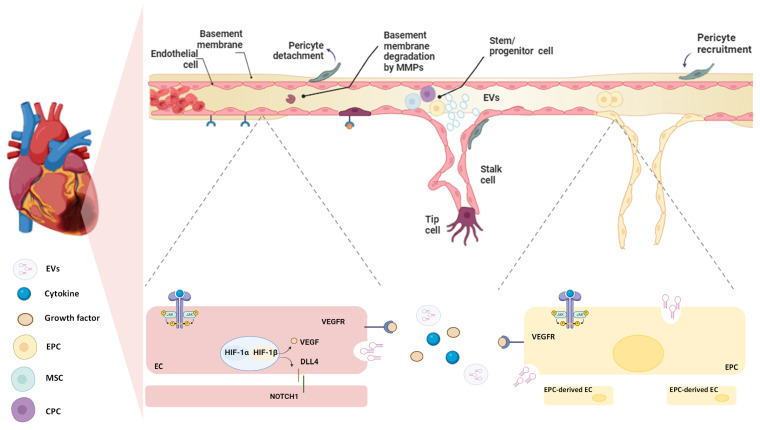
Vascular regeneration after MI. Hypoxia triggers vascular regeneration in the damaged heart tissue. Hypoxia Inducible factor (HIFs) stimulate the production of vascular endothelial growth factor (VEGF), which promotes the degradation of the basement membrane, allowing the migration of ECs and the recruitment of stem/precursor cells. In particular, endothelial progenitor cells (EPCs), cardiac progenitor cells (CPCs), and mesenchymal stem cells (MSCs) are recruited to the site of injury, where they release a variety of pro-angiogenic factors and miRNAs. These signals stimulate pre-existing endothelial cells (ECs) to differentiate into tip and stalk cells, which drive the sprouting of new blood vessels. Additionally, the EPCs themselves are stimulated to differentiate into mature ECs, contributing directly to the formation of new vasculature. These mechanisms are mainly regulated by the DLL4/Notch signalling pathway driven by cytokines such as interleukins (IL-6 and IL-10) (as described in Section 4.2. paragraph). In the final stages, pericytes are recruited to stabilise and mature the newly formed vessels, ensuring proper vascular function in the healing heart.

Angiogenesis is the process by which new blood vessels develop by sprouting from pre-existing vessels [165] (Figure 3). Angiogenesis is a dynamic process that involves interactions among ECs, ECM components, and signalling molecules released in the ischaemic area [166]. After MI, EC proliferation peaks within 4 days of infarction in the mouse heart and is mainly located at the interface of the infarct and viable tissue [167]. The beginning of sprouting angiogenesis occurs when oxygen-sensing mechanisms detect hypoxia. Studies conducted on mice and rats showed that the infarct core is characterised by severe hypoxia that stimulates EC proliferation and facilitates vascular expansion within the infarcted region, coordinated by HIFs [80,164]. HIFs stimulate the release of vascular endothelial growth factor-A (VEGF-A) by parenchymal cells, which plays a key role in guiding the growth of new blood vessels [168,169]. Angiogenesis involves several key steps and distinct EC phenotypes. First, the tip cells, which are specialised ECs, lead the vessel to sprout by migrating toward the source of VEGF-A. These tip cells extend long, thin filopodia that sense and respond to VEGF-A concentrations, allowing them to cross through the ECM toward the angiogenic stimulus, thus creating a path for the developing blood vessel. Closely behind the tip cells are the stalk cells, which are highly proliferative and responsible for elongating the nascent vessel. These cells form the trunk of the new capillary, eventually developing a lumen through which blood can flow. The coordinated action of tip and stalk cells ensures that the new vessel extends effectively toward the area of hypoxia. Once the new vessel is formed and perfused, the EC undergoes another transformation into quiescent phalanx cells. These cells acquire a cobblestone-like morphology, stabilising the vessel and maintaining its integrity. The final maturation and stabilisation of the capillary involve the recruitment of pericytes and the deposition of ECM [170,171] (Figure 3). EC proliferation can also be regulated by paracrine mediators and GFs that are released from cells located near the infarcted area [171]. In this regard, Dittrich et al. demonstrated the paracrine pro-angiogenic effect of Gata4/6+ FBs on ECs expansion and migration, highlighting that the double deletion of Gata4/6 transcription factors in FBs led to a reduction in capillary density within cardiac tissue and impaired the adaptive angiogenic response [172].

Arteriogenesis, instead, is the growth and enlargement of pre-existing collateral arterioles initiated by elevated shear stress on the vessel wall [161] that can take place in physiological (such as physical exercise) and pathological (such as MI) conditions [173]. It shares molecular mediators with angiogenesis, such as the induction of nitric oxide (NO), VEGF, and monocyte chemoattractant protein-1 (MCP-1), which increase in response to shear stress. The shear stress induces the entry of ECs into the cell cycle, facilitates monocyte migration across the endothelium, and promotes ECM remodelling, thereby supporting the development of a stable vascular network. This remodelling of pre-existing anastomotic collateral arteries increases both diameter and length, rather than an enhancement of collateral artery number [174]. This process, known as outward remodelling, significantly improves blood flow capacity after MI, thus allowing adequate tissue perfusion restoration to ischaemic areas that is necessary for cardiac function preservation [175]. Indeed, the presence of coronary collateralization is considered a significant predictor of long-term survival in individuals with coronary artery disease (CAD) [176].

On the contrary, vasculogenesis is characterised by de novo formation of capillaries by differentiation of multiple cell types, including EPCs and MSCs [165]. Although more prominently seen in early development, tissue ischaemia can also trigger postnatal vasculogenesis [177] (Figure 3).

### 3.1. Resident and/or Recruited Stem/Progenitor Cells in Vascular Regeneration

The role of stem/progenitor cells after MI is increasingly recognised as pivotal in promoting vascular regeneration. In addition to circulating stem/progenitor cells, which are recruited by ischaemic injury and contribute to endogenous neovascularisation, resident progenitor cells also play a role in repairing the cardiac vascular endothelium. These cells can differentiate into vascular components, influencing vascular remodelling, angiogenesis, and the inflammatory response following heart injury [178]. These cells can stimulate nearby ECs via paracrine action [51] through the secretion of factors and exosomes that help maintain vascular homeostasis and inhibit CM apoptosis, further supporting myocardial repair [179]. Understanding the mechanisms by which these endogenous cells enhance neovascularisation in injured regions offers valuable clinical insights for developing treatments for cardiovascular diseases and identifying potential contributors to cardiac regeneration [180].

#### 3.1.1. EPCs

Independent of their origin, it is widely agreed that EPCs display endothelial features and possess a natural affinity for vascular tissues, highlighting the inherent regenerative potential of the vascular system [181]. These EPCs will infiltrate the site of injury, where they can either differentiate into mature ECs or regulate pre-existing ECs via paracrine/juxtacrine signalling [182]. Mobilisation, migration, proliferation, and differentiation of EPCs orchestrate neovascularisation and re-endothelialization by regulating cytokines, receptors, adhesion molecules, proteases, and cellular signalling pathways [183].

The earliest evidence of circulating EPCs dates back to 1997, when researchers isolated CD34+ and Flk-1+ cells from peripheral blood capable of differentiating into ECs [184]. In the years following the study by Asahara and colleagues, numerous types of circulating EPCs have been identified. However, it was only in the last decade that researchers revealed the unique capability of CD34+ EPCs to promote neovasculogenesis during cardiac regeneration [185,186]. Circulating EPCs are recruited from the bone marrow to the ischaemic heart and can differentiate into ECs or SMCs, facilitating the development of new blood vessels [187]. The mobilisation of EPCs from the bone marrow occurs in response to elevated levels of circulating VEGF, which is highly released in response to hypoxia and inflammatory processes in MI [188,189]. In particular, it has been demonstrated that one of the mechanisms by which circulating EPCs induce angiogenesis is mediated through VEGFR-2, and EPCs with elevated VEGFR-2 levels exhibit a greater angiogenic capacity indeed [190]. Moreover, this study highlighted that EPC proliferation, angiogenesis-related gene expression levels, and vessel density were different among donors. Moreover, high concentrations of stem cell-derived factor-1 (SDF-1), also known as CXC motif chemokine 12, both in circulation and within the damaged tissue, contribute to the mobilisation of EPCs from the bone marrow. In particular, SDF-1 favours EPC recruitment to the site of injury, where its levels have been observed to be elevated from the 3rd day post-tissue damage onwards [191]. The onset of tissue regeneration aligns with a reduction in inflammation and an increase in anti-inflammatory cytokines, such as IL-10, which in turn promotes vascular network formation by significantly enhancing the concentration of EPCs at the wound site. Furthermore, an in vitro investigation performed by Short and co-workers revealed that EPC culture treated with an IL-10-conditioned medium effectively promotes endothelial sprouting and network formation [192]. In a murine model, the regenerative effect of exosomes isolated from wild-type EPCs and IL-10 KO EPCs was examined by injecting them into the myocardium immediately post-MI. Exosomes from wild-type EPCs improved neovascularisation and left ventricular cardiac function and significantly reduced CM apoptosis and the extension of infarct size, while EPC exosomes from the IL-10 KO group produced opposite effects [193]. In recent years, among the different types of exosomes derived from EPCs, those containing miRNA-126 play a key role in CM protection, neovascularisation, and tissue repair. Their release is conditioned by various external stimuli, such as hypoxia or inflammatory conditions, which also occur after MI [194].

The study conducted by Huang et al. underscored the significance of predominantly anti-inflammatory signalling in facilitating EPC-mediated myocardial revascularisation. These authors engineered exosomes from adipose tissue-derived stem cells (ADSCs) to overexpress Sirtuin 1 (SIRT1) and treated circulating EPCs isolated from MI patients, thus enhancing their cell migratory capacity. Moreover, in vivo administration of these exosomes in a mouse MI model led to increased angiogenesis within the infarcted tissue and improved cardiac function 28 days post-MI [195].

Two distinct subsets of EPCs have emerged for their roles in post-MI neovascularisation: myeloid angiogenic cells (MACs) and endothelial colony-forming cells (ECFCs). MACs are characterised by a relatively low proliferation rate, elevated CD34 levels, and diminished expression of stem cell-related markers CD133 and c-Kit [196]. Conversely, ECFCs exhibit robust proliferation, clonogenic potential, and substantial postnatal vascularization capacity also in the infarcted cardiac tissue [197]. Moreover, besides being CD34+, ECFCs express late endothelial differentiation genes including CD105, CD146, CD31, and VE-cadherin [198,199]. However, ECFCs have demonstrated diminished vascular regenerative capabilities in ischaemic settings, while enhanced angiogenesis has been observed when ECFCs were utilised in post-MI regenerative therapy in conjunction with MSCs [200].

In contrast, another recent study involving a limited participant cohort asserts that circulating EPCs responsible for cardiac neovascularisation did not originate from bone marrow but from the vessel wall [201]. The study enrolled male patients who received allogeneic bone marrow transplants from female donors, allowing the researchers to distinguish EPCs based on their genotype. The authors observed that ECs derived from the vascular wall were capable of proliferating in culture and forming a monolayer of CD31+ cells with an XY genotype, thus excluding that these cells originated from the bone marrow [201]. In line with these authors, there is also evidence suggesting the presence of resident EPCs in the heart. By using an EC-specific multispectral lineage-tracing mouse model (Pdgfb-iCreERT2-R26R-Brainbow2.1) combined with single-cell RNA sequencing, Li and colleagues demonstrated that a specific subset of resident EPCs actively contributes to the formation of new blood vessels in the adult mouse heart 7 days after MI [202]. Additionally, this study identified plasmalemma vesicle-associated protein (Plvap) as a novel endothelial-specific marker of cardiac neovasculogenesis, which was also identified in cardiac samples from patients with ischaemic heart disease [202]. The repair of damaged vessels by EPCs can occur through direct differentiation into ECs and integration into the injured vessels. However, an emerging feature of EPCs is their ability to act on cells and blood vessels through paracrine activity. EPCs release soluble pro-angiogenic factors, which directly or indirectly support the angiogenesis process by influencing migration, differentiation, mesenchymal to endothelial transition (MET), and integration of the differentiated cells as part of the intrinsic repair process [203]. EPCs also release EVs with a specific payload of pro-angiogenic miRNAs that regulate the molecular signalling pathways involved in neovascularisation [204].

The positive effects of an EPC-EV-based treatment were demonstrated in a rat model of MI [205]. The study highlighted the effectiveness of using a shear-thinning hydrogel to enhance EV delivery and retention in the ischaemic region. LAD occlusion was performed in rats, and then EPCs and EPC-EVs were injected into the border zone of the infarcted area. The results showed that both EPC and EPC-EV injections significantly improved vascular structure, cell proliferation, and haemodynamic function compared to the control group [205].

#### 3.1.2. CPCs

There is compelling evidence suggesting that c-Kit+ CPCs possess the ability to augment angiogenesis and attenuate myocardial fibrosis in rat models post-MI [206].

Since complete cardiac vascular regeneration cannot be achieved only through the direct differentiation of progenitor cells into mature cells, it has been demonstrated that these cells exert their regenerative potential through a paracrine mechanism mediated by EVs present in their secretome [51]. CPC-EVs are taken up by ECs, thus promoting angiogenesis through the delivery of both associated and co-isolated proteins [207]. These vesicles contain proangiogenic factors, including VEGF and FGF, as well as matrix proteins and integrins. CPC-EVs interact with ECs via endocytosis or membrane fusion, activating key signalling pathways such as PI3K/Akt and MAPK that enhance EC proliferation, migration, and survival. This results in improved vascular regeneration after myocardial injury by creating a favourable microenvironment for tissue repair [207]. Recent research has revealed that adult CPCs, however, exhibit a reduced regenerative capacity compared to neonatal CPCs [208]. For instance, adult CPCs lack the expression of YAP1, which is crucial for activating pro-regenerative and pro-survival pathways. This limitation was highlighted in a recent work where 72-h exposure of adult CPC clones to EVs derived from neonatal CPCs fosters the AKT signalling pathway, reflecting the well-established interplay between AKT and YAP1 in promoting cell proliferation and survival [208]. Vrijsen and colleagues pinpointed the role of CD147, also known as Extracellular Matrix Metalloproteinase Inducer (EMMPRIN), present in EVs of adult human CPC in mediating in vivo vascular regeneration through the stimulation of angiogenesis, as evidenced by the increase in CD31+ and alfa-SMA+ cells along with their co-localisation, typical of mature vessels [209]. In another study, human CPC-Exo were enriched in miR-210 and miR-132, which play important roles in angiogenesis and vascular remodelling. In a preclinical rat model of MI, CPC-Exo were able to promote angiogenesis and inhibit CM apoptosis, along with the improvement of cardiac function, through miR-132 and miR-210 [210]. miR-132 stimulates EC proliferation and migration by activating pro-angiogenic signalling pathways, thereby contributing to the formation of new blood vessels. On the other hand, miR-210 enhances cell survival under hypoxic conditions and regulates the cellular response to ischaemic stress while also promoting EC differentiation and angiogenesis [210]. Furthermore, CPC-Exo can also be engineered to deliver key molecules aimed at promoting cardiac neovascularization. An increase in capillary density has been observed in the infarcted area of the myocardium in mice following the administration of exosomes isolated from CPCs and bioengineered with a pro-angiogenic miR-322, delivered through the caudal vein [211].

#### 3.1.3. MSCs

One of the key mechanisms contributing to the beneficial outcomes of MSCs in cardiac vascular regeneration, akin to EPCs, is their paracrine activity [40,212]. The secretome of MSCs is believed to play a central role in both the initial phases of response to MI-induced damage, facilitating and sustaining the inflammatory process, and in the subsequent stages, aimed at attenuating inflammation and fostering an anti-inflammatory milieu (for an in-depth review, refer to Wagner et al., [213]). MSCs secrete a range of bioactive molecules, such as VEGF, HGF, and IGF1, and are shown to promote angiogenesis and cell survival, as well as reduce inflammation and inhibit apoptosis [214].

Among the recent evidence, Klopsch et al. demonstrated that CD45− CD44+ DDR2+ MSCs participate in the early stages of cardiac regeneration after MI [215]. Indeed, following the ischaemic event, both oxygen tension and paracrine activity of infiltrated polarised macrophages can regulate the MSC niche, promoting the expression of CD44 and DDR2 antigens. This study successfully identified tissue-specific ischaemia-responsive cardiac MSCs that possibly could take part in the regeneration of the ischaemic heart [215]. Subsequently, the same group demonstrated that co-culturing HUVECs with MSCs (CD45−CD44+DDR2+) significantly enhanced angiogenesis, leading to a roughly fourfold increase in the formation of branched and junctional networks, while unstimulated HUVECs exhibited only minimal angiogenic activity [216].

Moreover, MMPs are implicated in tissue revascularisation, regulating capillary diameter, and possibly stabilising nascent vessels after MI, highlighting the potential role of MMPs in angiogenesis after MI [80]. Other authors have demonstrated in a murine model of MI that the promotion of EC-induced microvascular regeneration is mediated by the MSC-derived SDF-1-enriched exosome, which stimulated MMP-2 and -9 protein levels [217]. Co-culturing human MSCs with ECs has led to the secretion of pro-angiogenic growth factors and matrix remodelling through the activation of MMP-2. MSC-EC crosstalk resulted in the formation of a rich vascular network with intercapillary distances similar to those observed in native myocardium [218]. In fact, it has been seen that the angiogenic potential of ECs could be enhanced by EMMPRIN, MM9, and VEGF contained in MSC-derived EVs, the key enzyme involved in promoting ECM remodelling and consequently supporting tissue regeneration in the myocardium [209]. The in vitro EMMPRIN-knockdown MSC-derived exosomes significantly reduced EC migration capacity with consequent almost complete inhibition of vessel formation, thus confirming the crucial role of EMMPRIN. These studies strengthen that MSCs can promote angiogenesis by modulating protease expression [209]. Endogenous c-Kit+ MSCs play a crucial role in enhancing capillary neovascularisation and improving cardiac function in mice with MI lesions. Specifically, Set domain-containing protein 4 (Setd4) epigenetically modulates the quiescence of c-Kit+ cells. In Setd4 knockout models, activated c-Kit+ cells have been observed, leading to improved cardiac function in mice with MI through capillary neovascularisation [180].

Transcription factors are pivotal molecules that govern gene expression, and, in the adult heart, GATA4 has a central role in progenitor cell signalling in cardiac repair and regeneration [219]. The analysis of mouse cardiac tissue at two, three, and four days after MI revealed that intravenous transplantation of GATA-4-overexpressing MSCs significantly increased the number of blood vessels and c-Kit+ cardiac cells [220]. The role of GATA-4 in promoting MSC-mediated cardiac vascular regeneration appears to be closely associated with the release of EVs [221]. In particular, exosomes derived from GATA-4-modified MSCs contain significant levels of the Let-7 miRNA family, which is known for its pro-angiogenic properties. Let-7 miRNAs regulate the expression of genes critical for EC proliferation, migration, and survival processes that are essential for new blood vessel formation [221]. The pro-angiogenic role of GATA-4-overexpressing MSC-derived exosomes has been further confirmed in a mouse MI model. A significant enhancement in vessel density was observed in the hearts of mice treated with GATA-4 MSC-derived exosomes 48 h after MI induction, compared to those treated with control MSC exosomes or the untreated MI groups [222].

## 4. Involved Pathways in Cardiac Regeneration

The proper development of the heart is coordinated by the action of multiple signalling pathways that, in response to specific stimuli, trigger the development and acquisition of cardiac tissue functionality. Among the various signalling pathways involved in these processes are the Hippo, Notch, Wnt, Hedgehog, and TGF-β superfamily pathways. All these processes act synergistically with transcription factors and regulators, aiming to control the proliferation and differentiation of cardiac precursors, contributing to heart formation [42]. In this section of the review, these signalling pathways are described, particularly focusing on their role in cardiac development and cardiac regeneration after MI.

### 4.1. Hippo Pathway

The Hippo pathway plays a central role in controlling various mechanisms of proliferation, survival, and differentiation of different cellular populations involved in tissue homeostasis and repair, including cardiac tissue [223]. 

Its main mechanism of action involves two transcription factors, Yes Associated Protein (YAP) and Transcriptional Co-Activator With PDZ-Binding Motif (TAZ), which are the main downstream effectors [224]. 

When the Hippo signalling pathway is active, multiple upstream signals regulate the phosphorylation of kinases MST1/MST2, LATS1/LATS2, and phosphorylate the proteins YAP/TAZ, which do not translocate into the nucleus and do not induce the transcription of target proteins. When the Hippo signalling pathway is inactive/off, YAP/TAZ are not phosphorylated; they localise in the nucleus, forming a complex that induces the transcription of genes necessary for cell proliferation, migration, and survival [225]. 

In mice, YAP is expressed during both foetal cardiac development and postnatal life, but its levels decrease in adulthood as its inhibitor Vestigial-like family member 4 (VGLL4) increases [226]. 

YAP activity may promote heart regeneration by multiple mechanisms, such as direct regulation of genes encoding proteins that control cytoskeletal dynamics and cell proliferation [227], stimulating IGF-1 and Akt signalling [228]. It has been demonstrated that during MI, the Hippo pathway is deactivated, resulting in specific activation of YAP in the damaged tissue, which promotes survival and proliferation of CMs [229]. Moreover, experiments involving overexpression of YAP in adult hearts show induction of cardiac regeneration and increased murine cardiac contractility after MI, providing further evidence of the role played by the Hippo pathway in tissue regeneration following damage [228]. By silencing certain components of the Hippo pathway, such as MST1 or Salv, there is an increase in the number of proliferating adult CMs, a heightened molecular reparative response, and improved ventricular regeneration in murine models of MI [230,231]. The ability of YAP to maintain the functionality of adult CMs and induce effective regeneration after tissue injury depends on its interaction with Paired-like homeodomain transcription factor 2 (Pitx2) [232]. These data confirm that the Hippo pathway is the main suppressor of cardiac proliferation and potential regeneration after MI, suggesting that YAP could be a potential therapeutic target to trigger endogenous cardiac regeneration.

### 4.2. Notch Pathway

The Notch signalling pathway is highly conserved across species and, in the past few decades, has been found to have a role in both development and tissue regeneration of multiple organs, such as the heart, hair cells, and the liver [233]. In mammals, Notch signalling acts through four receptors (Notch 1–4) and five ligands (Delta-like (DLL) 1, 3, 4, and Jagged 1, 2) triggering different cellular responses in healthy and pathological conditions via complex transduction mechanisms [234]. Notch involvement in angiogenesis, particularly in both normal and pathological conditions, remains an area of research. It controls key processes such as angiogenesis and endothelial sprouting, with its receptors and ligands expressed in ECs to maintain homeostasis and function [235]. The DLL4 signalling pathway is related to sprouting angiogenesis and artery formation. VEGF-A activates DLL4 of tip cells and transmits the signals to nearby ECs [236]. Signalling pathways such as VEGF-A and HIF-1α synergistically work together with Notch in post-infarction angiogenesis [237,238]. The administration of an adenovirus overexpressing the Notch intracellular domain (NICD) in rat infarcted hearts increased VEGF expression and angiogenesis, improving cardiac function [239]. EVs secreted by Notch1-overexpressing MSCs proved highly effective in preventing cell death, promoting angiogenesis, and CM proliferation, thus restoring cardiac function when injected in mice after MI [240]. Notch pathway also crosstalks with PI3K/Akt signalling pathways after MI. Indeed, it has been evidenced that Notch was activated by PI3K/Akt signalling, yet at the same time, Notch itself enhanced the expression of PI3K/Akt signalling in adult myocardium following MI, suggesting a positive survival feedback mechanism between Notch and Akt signalling [241]. Interestingly, the main downstream effectors of the Akt signalling pathway, such as eNOS, VEGF, mTOR, or FOXO, contribute to myocardial angiogenesis [242,243]. The initial evidence implicating the Notch signalling pathway in cardiac repair was demonstrated in the zebrafish model following partial ventricular apex amputation, where it was shown to be essential for CM proliferation and, consequently, for efficient cardiac repair after injury [244]. In mammals as well, components of the Notch signalling pathway are gradually upregulated during cardiac regeneration, highlighting its involvement in both embryonic and adult cardiogenesis [245]. Notch signalling is widely activated in CMs and mesenchymal cardiac precursors in murine models of cardiac hypertrophy and failure. Notch1 depletion impairs cardiac fibrosis and hypertrophy, suggesting its potential therapeutic relevance in mitigating adaptive hypertrophy after cardiac injury [246]. While inducing Notch signalling enhances CM proliferation in neonatal murine hearts post-injury, adult hearts remain unresponsive to Notch activation, failing to induce CM cell cycle re-entry, likely due to epigenetic modifications inhibiting Notch-responsive promoters [247]. Nemir et al. then demonstrated that Jagged1 overexpression in adult murine hearts subjected to pressure overload reduces FB proliferation while promoting the expansion of cardiac precursor cells [248]. These findings suggest a role for Notch signalling in modulating the balance between fibrotic and regenerative repair in the adult heart [249]. Inhibition of Notch signalling via deletion of recombination signal-binding protein-J (RBP-J) transcription factor leads to impaired CM apoptosis and reduced cardiac remodelling capacity post-MI in murine hearts, underscoring Notch signalling protective role against cell death following cardiac damage [250]. Collectively, this scientific evidence highlights the pivotal role of Notch signalling in heart regeneration and its complex interplay with various molecular pathways. For these reasons, Notch signalling is a highly conserved pathway during foetal and adult heart development that reduces cardiac scarring, improves cardiac function, and enhances CM proliferation [245]. However, it is necessary to elucidate the downstream effectors of Notch signalling and also identify the molecular mechanisms governing Notch activation in regenerating adult hearts.

### 4.3. Wnt/β-Catenin and Jak/Stat Pathways

#### 4.3.1. Wnt/β-Catenin

In addition to the widely studied classical pathways, Wnt/β-catenin plays a role in both embryonic development and adult tissue homeostasis regeneration. This pathway can be classified into two main types: canonical, known as Wnt/β-catenin, and non-canonical. In the canonical way, β-catenin translocates into the nucleus, leading to the activation of TCF/LEF transcription factors, primarily regulating cell proliferation. The non-canonical way operates independently of β-catenin and regulates cell polarity and migration [251]. The Wnt/β-catenin pathway is an important regulator of cardiac development and growth, and its activity in healthy adult hearts is low, essential for maintaining normal heart function. Acute activation of the Wnt/β-catenin pathway is thought to play a cardioprotective role after infarction through the upregulation of pro-survival genes and metabolic reprogramming [252]. Through the use of a reporter system in murine and rat models, the precise localisation of Wnt pathway activation following cardiac injury was identified specifically within the border zone of the infarcted region [253]. It has been reported that this pathway is gradually activated 24 h after MI and subsequently downregulated three weeks post-MI [254]. Recently, it has also been shown the direct activity of β-catenin in inducing mature CM proliferation after MI [255]. Reactivation of Wnt/β-catenin signalling enhances CM proliferation by increasing the nuclear import of β-catenin, which in turn activates the transcription of key target genes (such as *Wnt1, Wnt2, Wnt6, Axin2*, and *Lef1*). This process induces CM cytokinesis in both the infarct and border zones after MI, ultimately reducing infarct size and improving cardiac function [255]. Despite these findings demonstrating that the reactivation of the Wnt/β-catenin pathway leads to cardiac regeneration, as far back as 2008, indications emphasised that the silencing of β-catenin in KO mice resulted in increased differentiation of resident cardiac progenitor cells, suggesting that the downregulation of β-catenin was directly involved in endogenous cardiac regeneration [256]. These results were also recently confirmed by Hodgkinson et al., which demonstrated that inhibition of β-catenin induces the differentiation of c-Kit+ resident cells into CMs in a murine model of MI [257]. Therefore, the role of the Wnt/β-catenin pathway in inducing CM proliferation remains controversial.

Wnt signalling is triggered after MI injury and is implicated in cardiac regeneration, but it is also related to inflammation and angiogenesis [258]. Mice treated with the GSK-3β inhibitor (responsible for β-catenin degradation) displayed a robust activation of the Wnt/β-catenin signalling pathway with enhanced expression of VEGF. This overexpression led to a significant increase in CD31+ positive cells 7 and 14 days after MI, resulting in augmented capillary density and tissue healing [259]. High β-catenin expression could promote EMT, which might be an important source of angiogenesis and muscle fibre cells and play a significant role in tissue repair [260]. In effect, Wnt family protein levels (Wnt2, Wnt4, Wnt10b, and Wnt11) are increased 5 days following MI in murine actin+ perivascular SMCs and subepicardial ECs, apparently through EMT mechanism [254]. In addition, genetically enhanced Wnt10b expression in transgenic mouse CMs improved arterial formation and attenuated fibrosis in cardiac tissue after injury [261].

#### 4.3.2. JAK-STAT

The JAK-STAT signalling pathway plays a crucial role in maintaining cardiac homeostasis. Considerable attention has been directed towards understanding the biological and pathophysiological significance of the JAK-STAT signal in cardiac myocytes. Collecting evidence suggests that this signalling pathway is activated in cardiac myocytes by various cytokines, including IL-6, granulocyte colony-stimulating factor (G-CSF), leptin, and erythropoietin, among others. Activation of the JAK-STAT pathway in the heart promotes CM survival and enhances myocardial angiogenesis, thereby protecting the myocardium from pathological stresses [262]. There are four members in the JAK family (JAK1, JAK2, JAK3, and TYK2) and seven members in STAT (STAT1, STAT2, STAT3, STAT4, STAT5A, STAT5B, and STAT6) [263]. The first studies regarding the role of the JAK/STAT pathway in the cardiac tissue have primarily focused on the investigation of STAT1 and STAT3 [264]. For instance, the supernatant from necrotic primary CMs activates the JAK1-STAT1 pathway and promotes the nuclear translocation of c-Fos and NFκB p65 when simulating the MI microenvironment, reducing CM apoptosis under hypoxic conditions. However, silencing STAT1 diminishes the apoptosis induced by the necrotic supernatant [265]. Conversely to STAT1’s pro-apoptotic role, STAT3 demonstrates an anti-apoptotic effect [266]. These findings suggest a close association between the JAK/STAT pathway and the apoptotic response following MI, with STAT1 and STAT3 exhibiting opposite effects.

The involvement of the JAK/STAT pathway in neovascularisation during MI was investigated with STAT3, which plays a central role in the formation of blood vessels. Its activity has been investigated in STAT3 cardiac-specific KO mice that displayed decreased myocardial capillary density and increased susceptibility to injury [267]. Similarly, the administration of hyaluronic acid oligosaccharides into MI mice promoted neovessel formation and improved cardiac function as assessed 28 days post-intervention. These beneficial effects were associated with the activation of chemokines expression implicated in macrophage polarisation and the stimulation of MAPK and JAK/STAT signalling pathways for myocardial function reconstruction, as revealed by transcriptomic analyses [268]. These studies suggest that the JAK/STAT signalling pathway has a role in cardiac remodelling after cardiac infarction by controlling angiogenesis [269].

### 4.4. Hedgehog Pathway

Among the pathways active during embryogenesis and latent during adulthood, the Hedgehog (Hh) pathway stands out. This signalling pathway is involved in embryonic cardiac development and coronary vascular system formation and can be reactivated in response to tissue damage for tissue regeneration. The Hh pathway is capable of regulating both cell proliferation and differentiation [270]. Hh signalling components are present in adult cardiovascular tissues, although their activity is at a very low level. Pathological conditions such as ischaemia, reactivate Hh signalling [270]. Hh signalling can promote coronary neo-angiogenesis, reduce cardiac fibrosis, decrease cardiac apoptosis, and protect CMs death from MI, demonstrating its role in cardiac repair and regeneration [271]. The Hh ligands are paracrine factors that can modulate communication between cells. In vertebrates, three different ligands have been identified: Sonic Hedgehog (Shh), Desert Hedgehog (Dhh), and Indian Hedgehog (Ihh) [272]. Shh, specifically, is involved in cardiac regenerative response in mammals. In a murine model of MI, it has been observed that its activation is increased in the damaged areas of the heart, and its hyperactivation through the use of agonists leads to enhanced cardiac regeneration, increased cell proliferation, and the formation of new blood vessels in the damaged cardiac tissue [273]. Shh is the most widely expressed and mediates key processes also in neovascularisation in response to injury. Initial reports of the angiogenic effect of Shh implicated an indirect mechanism where Shh stimulated the secretion of angiogenic growth factors. It has been demonstrated in a mouse model that Shh mediates angiogenesis through stromal cells, supporting the hypothesis that its activation leads to a consequent secretion of angiogenic proteins and growth factors that is sufficient to trigger angiogenesis in ECs [274].

There is evidence of a strong overexpression of Shh in the adult mouse during myocardial ischaemia. Shh promotes angiogenesis by stimulating the proliferation and migration of ECs and FBs, thereby facilitating the formation of new blood vessels and restoring blood flow to ischaemic areas. Additionally, Shh contributes to the reduction of CM apoptosis and enhances cell survival through the activation of anti-apoptotic pathways [275].

### 4.5. TGF-β Superfamily

After MI, activation of endogenous TGF-β signalling pathways associated with cardiac repair has been extensively documented, modulating injurious, inflammatory, reparative, angiogenic, and fibrogenic responses [276]. The TGF-β superfamily comprises 33 members subdivided into various subfamilies, including TGF-β (TGF-β1, -β2, and -β3) and bone morphogenetic proteins (BMPs). In the adult mammalian heart, TGF-β is latent. Following MI, activated cells increase TGF-β reserves through de novo synthesis of its three isoforms, which may regulate the phenotype and function of all cell types involved in cardiac injury and repair [276]. In vitro studies investigated TGF-β1-mediated actions. Administration of the TGF-β inhibitor (SB-431542) robustly induces the proliferation of human iPS-derived CMs, if combined with a Wee1 inhibitor, and overexpression of CDK4 and Cyclin D1 [135]. On the other hand, in vivo experiments focused on the role of TGF-β receptor-activated signalling and explored pathways common to all three isoforms and to other members of the TGF-β superfamily [277]. The inhibition of TGF-β receptor 1 (TGFBR1) by SB-431542 reduced the number of proliferating CMs in zebrafish embryos. Furthermore, robust activation of TGF-β/SMAD3 signalling has been documented during zebrafish heart regeneration in adults, as evidenced by the upregulation of TGF ligands (TGF-β 1a, TGF-β 1b, TGF-β2, and TGF-β3), receptors (alk5a known as TGFBR1, and alk5b known as TGFBR1), and the downstream effector SMAD3 [278]. Additionally, inhibition of TGF-β/SMAD3 signalling reduces CM cell cycle activity and abolishes heart regeneration in adult zebrafish upon cardiac injury [279]. BMPs are multifunctional growth factors belonging to the TGF-β family that play a key role in multiple stages of cardiac development, including CM growth and differentiation from the mesoderm and ventricular trabeculation. BMP family members can be further classified into several subgroups, including BMP-2/-4, BMP-5/-6/-7 (OP-1)/-8, BMP-9/-10, and BMP-12/-13/-14 [280,281].

RNA sequencing of regenerating zebrafish hearts highlighted BMP signalling activation in the border zone of damaged myocardium, evidenced by BMP ligands (BMP2 and BMP7) and receptors (Bmpr1aa) expression, along with downstream SMAD mediators (Smad 1, 5, and 8) activation. Notably, BMP signalling plays a crucial role in injury-induced CM proliferation in fish. Specifically, a loss-of-function mutation in Bmpr1aa reduces both CM proliferation and heart regeneration [282]. Induction of widespread BMP2 expression in transgenic mouse myocardium revealed ectopic EMT, promoting CM proliferation and immaturity, highlighting BMP2 potential in expanding immature CMs [283]. In a 2016 study, BMP10 was shown to play an essential role in murine cardiac development [284]. Subsequently, BMP10 action in adult murine hearts was investigated by inducing its constitutive expression, which led to cardiac protection against cardiac damage. Researchers also identified BMP10 activity through activation of both SMAD and STAT3 pathways [285]. Further investigation into BMP10 action in a murine model of MI demonstrated its ability to induce CM proliferation via the MAPK pathway, leading to improved cardiac function and promoting tissue regeneration in infarcted hearts [286].

## 5. Current Therapeutical Approaches and Future Perspectives

Numerous experimental approaches have been developed by leveraging current knowledge of endogenous cardiac regeneration processes [43,287]. This understanding largely stems from studies of the mechanisms observed in neonatal mammals, where regenerative capacity is more pronounced [288]. However, despite many approaches being tested in large animal models, only a few have advanced to human trials [289]. For this reason, this review focuses on better highlighting the significant efforts made in recent years to understand the molecular pathways, cellular actors, and related paracrine effects involved in myocardial regeneration. This updated overview of recent evidence by cells and mechanisms involved in cardiac regenerative processes aims to facilitate the identification of more targeted therapeutic approaches.

Among the different therapeutic approaches, cell-based therapies, while promising, face significant challenges [290]. These include overcoming the host immune response thus eliminating the need for long-term immunosuppression, and improving the survival of transplanted cells in the ischaemic environment, which is typically hostile to cell engraftment. While many recent studies highlight the paracrine role of stem cells, as mentioned in this review, where secreted factors support regeneration by activating endogenous tissue repair mechanisms [42], other research focuses on their potential for direct differentiation into CMs or vascular tissue [291,292]. Within this framework, the concept of fusion of donor cells with host CMs has become less emphasised, as it is now considered relatively rare in the heart [293]. The direct differentiation moves alongside the growing interest in the experiments using iPSC-derived CMs and vascular cell precursors due to their ability to be partially differentiated yet toward cardiac phenotype and closely mimic native cell types. However, also in this case, concerns related to transplant rejection, together with tumorigenicity, long-term stability, and proper integration into host tissue, remain to be addressed [294,295].

Identifying the most appropriate cell type is crucial to minimising the risk of fatal arrhythmias, which can result from inadequate electrical and mechanical coupling between implanted cells and cardiac tissue. High-efficiency protocols for cell differentiation and transplantation must be developed to ensure the production of functional and safe cells for clinical use [296] to minimise the associated risks, such as uncontrolled cellular proliferation or teratogenic effects [297]. Some authors suggested that EVs can overcome the limitations of cell-based therapy by exploiting paracrine signalling as much more effective and safer [298,299].

In addition to cell-based therapy, induction of endogenous CM proliferation can be achieved with various strategies, including small molecules and non-coding RNAs (ncRNAs) [300].

For instance, the administration of NRG-1 as well as constitutive overexpression of its receptor ERBB2 leads to cardiac hypertrophy due to long-lasting stimulation [106,301]. Interestingly, this side effect was abolished using a transitory overexpression of this receptor [103]. For this reason, deeper preclinical studies are required to optimise the regulation of growth factors in order to avoid adverse effects.

The use of ncRNAs to regulate myocardial proliferation and regeneration also faces obstacles, with notable adverse effects observed in preclinical models. For example, miR-199a-3p, as discussed in this review, led to fatal arrhythmias in pigs due to uncontrolled ncRNA expression [92]. In contrast, inhibition of miR-132 via antisense oligonucleotides has shown early success and is currently in a Phase 1b clinical trial (NCT04045405) [302]. Similar to cell-based therapies, ncRNA therapies face challenges related to the host’s immune response. In this regard, engineered solutions, such as biocompatible nanoparticles for ncRNA encapsulation, could optimise targeted and controlled drug delivery [303].

In this context, engineered tissues composed of biomaterials and nanocarriers offer a promising strategy. Their potential lies in their ability to be constructed from biocompatible and biodegradable materials and their utility as vehicles for pharmacologically active molecules, potentially enhancing therapeutic efficacy while minimising systemic side effects. For instance, the above-mentioned nanocarriers can be engineered to perform tissue-specific drug targeting, enhancing efficacy while reducing systemic toxicity [304]. 

Among engineered constructs, not only nanoparticles but also patches have shown therapeutic benefits. In fact, acellularized and cellularized patches can represent an optimal solution for ventricular wall stabilisation during cardiac repair, as well as create a proper microenvironment for controlled cell proliferation and paracrine stimulation [305,306,307]. Studies on small animal models have highlighted the patches’ ability to promote the paracrine action of cells involved in the regenerative processes [308,309].

## 6. Conclusions

Endogenous cardiac regeneration following MI is a complex and highly regulated process that involves stem/progenitor cells and various molecular mechanisms. Although there are limitations in demonstrating the existence of resident stem cells in the heart with direct ability to differentiate into CMs, it is clear that certain populations of stem cells, both resident and recruited, activate key molecular mechanisms for myocardial and vascular regeneration after injury, primarily through paracrine activity. This has been confirmed in various animal models, where significant neovascularisation has been observed, mediated by the secretion of biomolecules from stem cells that activate pro-angiogenic and pro-proliferative pathways in the infarcted tissue.

On the other hand, several studies have shown that complete cardiac regeneration is possible in zebrafish and neonatal hearts, thus suggesting that specific molecular mechanisms responsible for this process may be present, albeit differently regulated, in adult mammals. The loss or increase of these specific regulators may enable adult CMs to re-enter the cell cycle, thereby reducing fibrosis and improving the functional response of the heart after infarction. Reactivating the cell cycle in CMs and extending the proliferation window, which is typically limited to the first days after birth, represents a critical point for initiating the regenerative response.

An area of great interest within the scientific community pertains to cell cycle-related proteins and cyclin-dependent kinases, along with non-coding RNAs, which promote the re-entry of CMs into the cell cycle and their subsequent proliferation. These key molecules in the regenerative process could potentially be activated or inhibited in the adult model to stimulate the proliferation of existing CMs in damaged tissue and guide tissue regeneration.

Considerable knowledge has accumulated regarding the regulation of new vessel formation in healthy and infarcted hearts. It has become clear that ECs can respond to mechanical and paracrine stimuli from blood flow and neighbouring cells, including precursor cells, but also actively participate in cardiac remodelling processes. In adult hearts, precursors may also contribute to revascularisation, consistent with the concept of neovasculogenesis. The presented signalling pathways involved in neovascularisation represent promising therapeutic targets to improve cardiac remodelling and prevent HF development.

In conclusion, despite significant progress in the study of cardiac regeneration, it remains a complex challenge. Understanding the mechanisms underlying the proliferation of adult CMs, exploring new therapeutic targets, and developing advanced technologies for targeted therapy delivery are fundamental priorities in cardiovascular research. Only through an integrated approach that encompasses myocardial and vascular reconstruction, the activation of multiple signalling pathways, and the maintenance of tissue homeostasis is it possible to advance toward effective regenerative therapies for the infarcted heart.

## References

[1] Vaduganathan M., Mensah G.A., Turco J.V., Fuster V., Roth G.A. (2022). The Global Burden of Cardiovascular Diseases and Risk. J. Am. Coll. Cardiol..

[2] Litviňuková M., Talavera-López C., Maatz H., Reichart D., Worth C.L., Lindberg E.L., Kanda M., Polanski K., Heinig M., Lee M. (2020). Cells of the Adult Human Heart. Nature.

[3] Saleh M., Ambrose J.A. (2018). Understanding Myocardial Infarction. F1000Research.

[4] Buja L.M. (2023). Pathobiology of Myocardial Ischemia and Reperfusion Injury: Models, Modes, Molecular Mechanisms, Modulation, and Clinical Applications. Cardiol. Rev..

[5] Gabriel-Costa D. (2018). The Pathophysiology of Myocardial Infarction-Induced Heart Failure. Pathophysiology.

[6] Lusis A.J. (2000). Atherosclerosis. Nature.

[7] Baaten C.C., Nagy M., Bergmeier W., Spronk H.M.H., Van Der Meijden P.E.J. (2024). Platelet Biology and Function: Plaque Erosion vs. Rupture. Eur. Heart J..

[8] Rafieian-Kopaei M., Setorki M., Doudi M., Baradaran A., Nasri H. (2014). Atherosclerosis: Process, Indicators, Risk Factors and New Hopes. Int. J. Prev. Med..

[9] Yap J., Irei J., Lozano-Gerona J., Vanapruks S., Bishop T., Boisvert W.A. (2023). Macrophages in Cardiac Remodelling after Myocardial Infarction. Nat. Rev. Cardiol..

[10] Burgon P.G., Weldrick J.J., Talab O.M.S.A., Nadeer M., Nomikos M., Megeney L.A. (2023). Regulatory Mechanisms That Guide the Fetal to Postnatal Transition of Cardiomyocytes. Cells.

[11] Hinderer S., Schenke-Layland K. (2019). Cardiac Fibrosis—A Short Review of Causes and Therapeutic Strategies. Adv. Drug Deliv. Rev..

[12] Şabanoğlu C., Sinan Ü.Y., Akboğa M.K., Çoner A., Gök G., Kocabaş U., Bekar L., Gazi E., Cengiz M., Kılıç S. (2023). Long-Term Prognosis of Patients with Heart Failure: Follow-Up Results of Journey HF-TR Study Population. Anatol. J. Cardiol..

[13] Cluitmans M.J.M., Bayer J., Bear L.R., ter Bekke R.M.A., Heijman J., Coronel R., Volders P.G.A. (2023). The Circle of Reentry: Characteristics of Trigger-Substrate Interaction Leading to Sudden Cardiac Arrest. Front. Cardiovasc. Med..

[14] Sigamani A., Gupta R. (2022). Revisiting Secondary Prevention in Coronary Heart Disease. Indian Heart J..

[15] Sachdeva P., Kaur K., Fatima S., Mahak F., Noman M., Siddenthi S.M., Surksha M.A., Munir M., Fatima F., Sultana S.S. (2023). Advancements in Myocardial Infarction Management: Exploring Novel Approaches and Strategies. Cureus.

[16] Garbern J.C., Lee R.T. (2022). Heart Regeneration: 20 Years of Progress and Renewed Optimism. Dev. Cell.

[17] de Wit L., Fang J., Neef K., Xiao J., Doevendans P.A., Schiffelers R.M., Lei Z., Sluijter J.P.G. (2020). Cellular and Molecular Mechanism of Cardiac Regeneration: A Comparison of Newts, Zebrafish, and Mammals. Biomolecules.

[18] Costa A., Cushman S., Haubner B.J., Derda A.A., Thum T., Bär C. (2022). Neonatal Injury Models: Integral Tools to Decipher the Molecular Basis of Cardiac Regeneration. Basic Res. Cardiol..

[19] Aslan G.S., Polat F., Eren S.N., Yucel D., Arbatli S., Cumbul A., Kocabas F. (2021). Identification of Novel and Potent Modulators Involved in Neonatal Cardiac Regeneration. Pediatr. Cardiol..

[20] Notari M., Ventura-Rubio A., Bedford-Guaus S.J., Jorba I., Mulero L., Navajas D., Martí M., Raya Á. (2018). The local microenvironment limits the regenerative potential of the mouse neonatal heart. Sci. Adv..

[21] Shi X., Qiu H. (2022). New Insights into Energy Substrate Utilization and Metabolic Remodeling in Cardiac Physiological Adaption. Front. Physiol..

[22] Paltzer W.G., Aballo T.J., Bae J., Hubert K.A., Nuttall D.J., Perry C., Wanless K.N., Nahlawi R., Ge Y., Mahmoud A.I. (2023). MTORC1 Regulates the Metabolic Switch of Postnatal Cardiomyocytes During Regeneration. bioRxiv.

[23] Hashimoto H., Olson E.N., Bassel-Duby R. (2018). Therapeutic Approaches for Cardiac Regeneration and Repair. Nat. Rev. Cardiol..

[24] Wang Z., Cui M., Shah A.M., Ye W., Tan W., Min Y.L., Botten G.A., Shelton J.M., Liu N., Bassel-Duby R. (2019). Mechanistic Basis of Neonatal Heart Regeneration Revealed by Transcriptome and Histone Modification Profiling. Proc. Natl. Acad. Sci. USA.

[25] Wang Z., Cui M., Shah A.M., Tan W., Liu N., Bassel-Duby R., Olson E.N. (2020). Cell-Type-Specific Gene Regulatory Networks Underlying Murine Neonatal Heart Regeneration at Single-Cell Resolution. Cell Rep..

[26] Quaife-Ryan G.A., Sim C.B., Ziemann M., Kaspi A., Rafehi H., Ramialison M., El-Osta A., Hudson J.E., Porrello E.R. (2017). Multicellular Transcriptional Analysis of Mammalian Heart Regeneration. Circulation.

[27] Lock M.C., Darby J.R.T., Soo J.Y., Brooks D.A., Perumal S.R., Selvanayagam J.B., Seed M., Macgowan C.K., Porrello E.R., Tellam R.L. (2019). Differential Response to Injury in Fetal and Adolescent Sheep Hearts in the Immediate Post-Myocardial Infarction Period. Front. Physiol..

[28] Locatelli P., Belaich M.N., López A.E., Olea F.D., Uranga Vega M., Giménez C.S., Simonin J.A., Bauzá M.d.R., Castillo M.G., Cuniberti L.A. (2020). Novel Insights into Cardiac Regeneration Based on Differential Fetal and Adult Ovine Heart Transcriptomic Analysis. Am. J. Physiol.-Heart Circ. Physiol..

[29] Zhu W., Zhang E., Zhao M., Chong Z., Fan C., Tang Y., Hunter J.D., Borovjagin A.V., Walcott G.P., Chen J.Y. (2018). Regenerative Potential of Neonatal Porcine Hearts. Circulation.

[30] Mollova M., Bersell K., Walsh S., Savla J., Das L.T., Park S.-Y., Silberstein L.E., dos Remedios C.G., Graham D., Colan S. (2013). Cardiomyocyte Proliferation Contributes to Heart Growth in Young Humans. Proc. Natl. Acad. Sci. USA.

[31] Haubner B.J., Schneider J., Schweigmann U., Schuetz T., Dichtl W., Velik-Salchner C., Stein J.-I., Penninger J.M. (2016). Functional Recovery of a Human Neonatal Heart After Severe Myocardial Infarction. Circ. Res..

[32] Bergmann O., Bhardwaj R.D., Bernard S., Zdunek S., Barnabé-Heide F., Walsh S., Zupicich J., Alkass K., Buchholz B.A., Druid H. (2009). Evidence for Cardiomyocyte Renewal in Humans. Science.

[33] Singam N.S.V., Fine C., Fleg J.L. (2020). Cardiac Changes Associated with Vascular Aging. Clin. Cardiol..

[34] Xu J., Zuo C. (2021). The Fate Status of Stem Cells in Diabetes and Its Role in the Occurrence of Diabetic Complications. Front. Mol. Biosci..

[35] Molinaro C., Salerno L., Marino F., Scalise M., Salerno N., Pagano L., De Angelis A., Cianflone E., Torella D., Urbanek K. (2022). Unraveling and Targeting Myocardial Regeneration Deficit in Diabetes. Antioxidants.

[36] Moiseeva V., Cisneros A., Sica V., Deryagin O., Lai Y., Jung S., Andrés E., An J., Segalés J., Ortet L. (2023). Senescence Atlas Reveals an Aged-like Inflamed Niche That Blunts Muscle Regeneration. Nature.

[37] Hariharan N., Sussman M.A. (2015). Cardiac Aging—Getting to the Stem of the Problem. J. Mol. Cell. Cardiol..

[38] Yadav S.K., Kambis T.N., Kar S., Park S.Y., Mishra P.K. (2020). MMP9 Mediates Acute Hyperglycemia-Induced Human Cardiac Stem Cell Death by Upregulating Apoptosis and Pyroptosis in Vitro. Cell Death Dis..

[39] Guo J., Huang X., Dou L., Yan M., Shen T., Tang W., Li J. (2022). Aging and Aging-Related Diseases: From Molecular Mechanisms to Interventions and Treatments. Signal Transduct. Target. Ther..

[40] Cai M., Shen R., Song L., Lu M., Wang J., Zhao S., Tang Y., Meng X., Li Z., He Z.-X. (2016). Bone Marrow Mesenchymal Stem Cells (BM-MSCs) Improve Heart Function in Swine Myocardial Infarction Model through Paracrine Effects. Sci. Rep..

[41] Shen H., Gan P., Wang K., Darehzereshki A., Wang K., Kumar S.R., Lien C.-L., Patterson M., Tao G., Sucov H.M. (2020). Mononuclear Diploid Cardiomyocytes Support Neonatal Mouse Heart Regeneration in Response to Paracrine IGF2 Signaling. Elife.

[42] Witman N., Zhou C., Grote Beverborg N., Sahara M., Chien K.R. (2020). Cardiac Progenitors and Paracrine Mediators in Cardiogenesis and Heart Regeneration. Semin. Cell Dev. Biol..

[43] Wang J., An M., Haubner B.J., Penninger J.M. (2023). Cardiac Regeneration: Options for Repairing the Injured Heart. Front. Cardiovasc. Med..

[44] Chang N.C. (2020). Autophagy and Stem Cells: Self-Eating for Self-Renewal. Front. Cell Dev. Biol..

[45] Hesse M., Fleischmann B.K., Kotlikoff M.I. (2014). Concise Review: The Role of C-Kit Expressing Cells in Heart Repair at the Neonatal and Adult Stage. Stem Cells.

[46] Beltrami A.P., Barlucchi L., Torella D., Baker M., Limana F., Chimenti S., Kasahara H., Rota M., Musso E., Urbanek K. (2003). Adult Cardiac Stem Cells Are Multipotent and Support Myocardial Regeneration. Cell.

[47] Chien K.R., Frisén J., Fritsche-Danielson R., Melton D.A., Murry C.E., Weissman I.L. (2019). Regenerating the Field of Cardiovascular Cell Therapy. Nat. Biotechnol..

[48] van Berlo J.H., Molkentin J.D. (2016). Most of the Dust Has Settled. Circ. Res..

[49] Aquila I., Marino F., Cianflone E., Marotta P., Torella M., Mollace V., Indolfi C., Nadal-Ginard B., Torella D. (2018). The Use and Abuse of Cre/Lox Recombination to Identify Adult Cardiomyocyte Renewal Rate and Origin. Pharmacol. Res..

[50] Gude N.A., Firouzi F., Broughton K.M., Ilves K., Nguyen K.P., Payne C.R., Sacchi V., Monsanto M.M., Casillas A.R., Khalafalla F.G. (2018). Cardiac C-Kit Biology Revealed by Inducible Transgenesis. Circ. Res..

[51] Bryl R., Kulus M., Bryja A., Domagała D., Mozdziak P., Antosik P., Bukowska D., Zabel M., Dzięgiel P., Kempisty B. (2024). Cardiac Progenitor Cell Therapy: Mechanisms of Action. Cell Biosci..

[52] Le T., Chong J. (2016). Cardiac Progenitor Cells for Heart Repair. Cell Death Discov..

[53] Cianflone E., Aquila I., Scalise M., Marotta P., Torella M., Nadal-Ginard B., Torella D. (2018). Molecular Basis of Functional Myogenic Specification of Bona Fide Multipotent Adult Cardiac Stem Cells. Cell Cycle.

[54] Barreto S., Hamel L., Schiatti T., Yang Y., George V. (2019). Cardiac Progenitor Cells from Stem Cells: Learning from Genetics and Biomaterials. Cells.

[55] Van Berlo J.H., Kanisicak O., Maillet M., Vagnozzi R.J., Karch J., Lin S.C.J., Middleton R.C., Marbán E., Molkentin J.D. (2014). C-Kit^+^ Cells Minimally Contribute Cardiomyocytes to the Heart. Nature.

[56] Vicinanza C., Aquila I., Scalise M., Cristiano F., Marino F., Cianflone E., Mancuso T., Marotta P., Sacco W., Lewis F.C. (2017). Adult Cardiac Stem Cells Are Multipotent and Robustly Myogenic: C-Kit Expression Is Necessary but Not Sufficient for Their Identification. Cell Death Differ..

[57] Ellison G.M., Torella D., Dellegrottaglie S., Perez-Martinez C., Perez de Prado A., Vicinanza C., Purushothaman S., Galuppo V., Iaconetti C., Waring C.D. (2011). Endogenous Cardiac Stem Cell Activation by Insulin-Like Growth Factor-1/Hepatocyte Growth Factor Intracoronary Injection Fosters Survival and Regeneration of the Infarcted Pig Heart. J. Am. Coll. Cardiol..

[58] He L., Nguyen N.B., Ardehali R., Zhou B. (2020). Heart Regeneration by Endogenous Stem Cells and Cardiomyocyte Proliferation. Circulation.

[59] Valiente-Alandi I., Albo-Castellanos C., Herrero D., Sanchez I., Bernad A. (2016). Bmi1^+^ Cardiac Progenitor Cells Contribute to Myocardial Repair Following Acute Injury. Stem Cell Res. Ther..

[60] Song Y., Zhao M., Xie Y., Zhu T., Liang W., Sun B., Liu W., Wu L., Lu G., Li T. (2019). Bmi-1 High-expressing Cells Enrich Cardiac Stem/Progenitor Cells and Respond to Heart Injury. J. Cell. Mol. Med..

[61] Soonpaa M.H., Lafontant P.J., Reuter S., Scherschel J.A., Srour E.F., Zaruba M.-M., Rubart-von der Lohe M., Field L.J. (2018). Absence of Cardiomyocyte Differentiation Following Transplantation of Adult Cardiac-Resident Sca-1^+^ Cells into Infarcted Mouse Hearts. Circulation.

[62] Vagnozzi R.J., Sargent M.A., Lin S.-C.J., Palpant N.J., Murry C.E., Molkentin J.D. (2018). Genetic Lineage Tracing of Sca-1^+^ Cells Reveals Endothelial but Not Myogenic Contribution to the Murine Heart. Circulation.

[63] Zhang L., Sultana N., Yan J., Yang F., Chen F., Chepurko E., Yang F.-C., Du Q., Zangi L., Xu M. (2018). Cardiac Sca-1^+^ Cells Are Not Intrinsic Stem Cells for Myocardial Development, Renewal, and Repair. Circulation.

[64] Neidig L.E., Weinberger F., Palpant N.J., Mignone J., Martinson A.M., Sorensen D.W., Bender I., Nemoto N., Reinecke H., Pabon L. (2018). Evidence for Minimal Cardiogenic Potential of Stem Cell Antigen 1–Positive Cells in the Adult Mouse Heart. Circulation.

[65] Tang J., Li Y., Huang X., He L., Zhang L., Wang H., Yu W., Pu W., Tian X., Nie Y. (2018). Fate Mapping of Sca1^+^ Cardiac Progenitor Cells in the Adult Mouse Heart. Circulation.

[66] Herrero D., Cañón S., Pelacho B., Salvador-Bernáldez M., Aguilar S., Pogontke C., Carmona R.M., Salvador J.M., Perez-Pomares J.M., Klein O.D. (2018). Bmi1-Progenitor Cell Ablation Impairs the Angiogenic Response to Myocardial Infarction. Arterioscler. Thromb. Vasc. Biol..

[67] Herrero D., Cañón S., Albericio G., Carmona R.M., Aguilar S., Mañes S., Bernad A. (2019). Age-Related Oxidative Stress Confines Damage-Responsive Bmi1+ Cells to Perivascular Regions in the Murine Adult Heart. Redox. Biol..

[68] Christoffels V., Jensen B. (2020). Cardiac Morphogenesis: Specification of the Four-Chambered Heart. Cold Spring Harb. Perspect. Biol..

[69] Liu Y., Chen L., Diaz A.D., Benham A., Xu X., Wijaya C.S., Fa’ak F., Luo W., Soibam B., Azares A. (2016). Mesp1 Marked Cardiac Progenitor Cells Repair Infarcted Mouse Hearts. Sci. Rep..

[70] Lee C.-S., Cho H.-J., Lee J.-W., Son H., Chai J., Kim H.-S. (2021). Adhesion GPCR Latrophilin-2 Specifies Cardiac Lineage Commitment through CDK5, Src, and P38MAPK. Stem Cell Rep..

[71] Bondue A., Tännler S., Chiapparo G., Chabab S., Ramialison M., Paulissen C., Beck B., Harvey R., Blanpain C. (2011). Defining the Earliest Step of Cardiovascular Progenitor Specification during Embryonic Stem Cell Differentiation. J. Cell Biol..

[72] Kattman S.J., Huber T.L., Keller G.M. (2006). Multipotent Flk-1^+^ Cardiovascular Progenitor Cells Give Rise to the Cardiomyocyte, Endothelial, and Vascular Smooth Muscle Lineages. Dev. Cell.

[73] Yang L., Soonpaa M.H., Adler E.D., Roepke T.K., Kattman S.J., Kennedy M., Henckaerts E., Bonham K., Abbott G.W., Linden R.M. (2008). Human Cardiovascular Progenitor Cells Develop from a KDR^+^ Embryonic-Stem-Cell-Derived Population. Nature.

[74] Bollini S., Smits A.M., Balbi C., Lazzarini E., Ameri P. (2018). Triggering Endogenous Cardiac Repair and Regeneration via Extracellular Vesicle-Mediated Communication. Front. Physiol..

[75] Gregory C.D., Rimmer M.P. (2023). Extracellular Vesicles Arising from Apoptosis: Forms, Functions, and Applications. J. Pathol..

[76] Farahzadi R., Fathi E., Valipour B., Ghaffary S. (2024). Stem Cells-Derived Exosomes as Cardiac Regenerative Agents. IJC Heart Vasc..

[77] Streef T.J., Smits A.M. (2021). Epicardial Contribution to the Developing and Injured Heart: Exploring the Cellular Composition of the Epicardium. Front. Cardiovasc. Med..

[78] Smits A.M., Dronkers E., Goumans M.-J. (2018). The Epicardium as a Source of Multipotent Adult Cardiac Progenitor Cells: Their Origin, Role and Fate. Pharmacol. Res..

[79] del Campo C.V., Liaw N.Y., Gunadasa-Rohling M., Matthaei M., Braga L., Kennedy T., Salinas G., Voigt N., Giacca M., Zimmermann W.-H. (2022). Regenerative Potential of Epicardium-Derived Extracellular Vesicles Mediated by Conserved MiRNA Transfer. Cardiovasc. Res..

[80] Li J., Zhao Y., Zhu W. (2021). Targeting Angiogenesis in Myocardial Infarction: Novel Therapeutics (Review). Exp. Ther. Med..

[81] Correia C.D., Ferreira A., Fernandes M.T., Silva B.M., Esteves F., Leitão H.S., Bragança J., Calado S.M. (2023). Human Stem Cells for Cardiac Disease Modeling and Preclinical and Clinical Applications—Are We on the Road to Success?. Cells.

[82] Bakinowska E., Kiełbowski K., Boboryko D., Bratborska A.W., Olejnik-Wojciechowska J., Rusiński M., Pawlik A. (2024). The Role of Stem Cells in the Treatment of Cardiovascular Diseases. Int. J. Mol. Sci..

[83] Joladarashi D., Kishore R. (2022). Mesenchymal Stromal Cell Exosomes in Cardiac Repair. Curr. Cardiol. Rep..

[84] Bagno L., Hatzistergos K.E., Balkan W., Hare J.M. (2018). Mesenchymal Stem Cell-Based Therapy for Cardiovascular Disease: Progress and Challenges. Mol. Ther..

[85] Bartczak A., McGilvray I., Keating A. (2017). Mesenchymal Stromal Cell Therapy to Promote Cardiac Tissue Regeneration and Repair. Curr. Opin. Organ Trans..

[86] Shao L., Zhang Y., Lan B., Wang J., Zhang Z., Zhang L., Xiao P., Meng Q., Geng Y., Yu X. (2017). MiRNA-Sequence Indicates That Mesenchymal Stem Cells and Exosomes Have Similar Mechanism to Enhance Cardiac Repair. Biomed Res. Int..

[87] Sun S.-J., Wei R., Li F., Liao S.-Y., Tse H.-F. (2021). Mesenchymal Stromal Cell-Derived Exosomes in Cardiac Regeneration and Repair. Stem Cell Rep..

[88] Hade M.D., Suire C.N., Suo Z. (2021). Mesenchymal Stem Cell-Derived Exosomes: Applications in Regenerative Medicine. Cells.

[89] Ferguson S.W., Wang J., Lee C.J., Liu M., Neelamegham S., Canty J.M., Nguyen J. (2018). The MicroRNA Regulatory Landscape of MSC-Derived Exosomes: A Systems View. Sci. Rep..

[90] Scott S.R., March K.L., Wang I.-W., Singh K., Liu J., Turrentine M., Sen C.K., Wang M. (2022). Bone Marrow- or Adipose-Mesenchymal Stromal Cell Secretome Preserves Myocardial Transcriptome Profile and Ameliorates Cardiac Damage Following Ex Vivo Cold Storage. J. Mol. Cell. Cardiol..

[91] Eulalio A., Mano M., Ferro M.D., Zentilin L., Sinagra G., Zacchigna S., Giacca M. (2012). Functional Screening Identifies MiRNAs Inducing Cardiac Regeneration. Nature.

[92] Gabisonia K., Prosdocimo G., Aquaro G.D., Carlucci L., Zentilin L., Secco I., Ali H., Braga L., Gorgodze N., Bernini F. (2019). MicroRNA Therapy Stimulates Uncontrolled Cardiac Repair after Myocardial Infarction in Pigs. Nature.

[93] Cheng H., Chang S., Xu R., Chen L., Song X., Wu J., Qian J., Zou Y., Ma J. (2020). Hypoxia-Challenged MSC-Derived Exosomes Deliver MiR-210 to Attenuate Post-Infarction Cardiac Apoptosis. Stem Cell Res. Ther..

[94] Arif M., Pandey R., Alam P., Jiang S., Sadayappan S., Paul A., Ahmed R.P.H. (2017). MicroRNA-210-Mediated Proliferation, Survival, and Angiogenesis Promote Cardiac Repair Post Myocardial Infarction in Rodents. J. Mol. Med..

[95] Yan W., Abu-El-Rub E., Saravanan S., Kirshenbaum L.A., Arora R.C., Dhingra S. (2019). Inflammation in Myocardial Injury: Mesenchymal Stem Cells as Potential Immunomodulators. Am. J. Physiol.-Heart Circ. Physiol..

[96] Jiang W., Wang M. (2019). New Insights into the Immunomodulatory Role of Exosomes in Cardiovascular Disease. Rev. Cardiovasc. Med..

[97] Zhao J., Li X., Hu J., Chen F., Qiao S., Sun X., Gao L., Xie J., Xu B. (2019). Mesenchymal Stromal Cell-Derived Exosomes Attenuate Myocardial Ischaemia-Reperfusion Injury through MiR-182-Regulated Macrophage Polarization. Cardiovasc. Res..

[98] Shen D., He Z. (2021). Mesenchymal Stem Cell-Derived Exosomes Regulate the Polarization and Inflammatory Response of Macrophages via MiR-21-5p to Promote Repair after Myocardial Reperfusion Injury. Ann. Transl. Med..

[99] Ren X., Zhao M., Lash B., Martino M.M., Julier Z. (2020). Growth Factor Engineering Strategies for Regenerative Medicine Applications. Front. Bioeng. Biotechnol..

[100] Payan S.M., Hubert F., Rochais F. (2020). Cardiomyocyte Proliferation, a Target for Cardiac Regeneration. Biochim. Biophys. Acta (BBA)—Mol. Cell Res..

[101] Wang Y., Wei J., Zhang P., Zhang X., Wang Y., Chen W., Zhao Y., Cui X. (2022). Neuregulin-1, a Potential Therapeutic Target for Cardiac Repair. Front. Pharmacol..

[102] Vermeulen Z., Segers V.F.M., De Keulenaer G.W. (2016). ErbB2 Signaling at the Crossing between Heart Failure and Cancer. Basic Res. Cardiol..

[103] Polizzotti B.D., Ganapathy B., Walsh S., Choudhury S., Ammanamanchi N., Bennett D.G., dos Remedios C.G., Haubner B.J., Penninger J.M., Kühn B. (2015). Neuregulin Stimulation of Cardiomyocyte Regeneration in Mice and Human Myocardium Reveals a Therapeutic Window. Sci. Transl. Med..

[104] Bae J., Paltzer W.G., Mahmoud A.I. (2021). The Role of Metabolism in Heart Failure and Regeneration. Front. Cardiovasc. Med..

[105] Honkoop H., de Bakker D.E., Aharonov A., Kruse F., Shakked A., Nguyen P.D., de Heus C., Garric L., Muraro M.J., Shoffner A. (2019). Single-Cell Analysis Uncovers That Metabolic Reprogramming by ErbB2 Signaling Is Essential for Cardiomyocyte Proliferation in the Regenerating Heart. eLife.

[106] D’Uva G., Aharonov A., Lauriola M., Kain D., Yahalom-Ronen Y., Carvalho S., Weisinger K., Bassat E., Rajchman D., Yifa O. (2015). ERBB2 Triggers Mammalian Heart Regeneration by Promoting Cardiomyocyte Dedifferentiation and Proliferation. Nat. Cell Biol..

[107] Aharonov A., Shakked A., Umansky K.B., Savidor A., Genzelinakh A., Kain D., Lendengolts D., Revach O.Y., Morikawa Y., Dong J. (2020). ERBB2 Drives YAP Activation and EMT-like Processes during Cardiac Regeneration. Nat. Cell Biol..

[108] Zhu Y., Do V.D., Richards A.M., Foo R. (2021). What We Know about Cardiomyocyte Dedifferentiation. J. Mol. Cell. Cardiol..

[109] Youssef K.K., Nieto M.A. (2024). Epithelial–Mesenchymal Transition in Tissue Repair and Degeneration. Nat. Rev. Mol. Cell Biol..

[110] Khosravi F., Ahmadvand N., Bellusci S., Sauer H. (2021). The Multifunctional Contribution of FGF Signaling to Cardiac Development, Homeostasis, Disease and Repair. Front. Cell Dev. Biol..

[111] Prudovsky I. (2021). Cellular Mechanisms of FGF-Stimulated Tissue Repair. Cells.

[112] Bongiovanni C., Sacchi F., Pra S.D., Pantano E., Miano C., Morelli M.B., D’uva G. (2021). Reawakening the Intrinsic Cardiac Regenerative Potential: Molecular Strategies to Boost Dedifferentiation and Proliferation of Endogenous Cardiomyocytes. Front. Cardiovasc. Med..

[113] Itoh N., Ohta H., Nakayama Y., Konishi M. (2016). Roles of FGF Signals in Heart Development, Health, and Disease. Front. Cell Dev. Biol..

[114] Formiga F.R., Pelacho B., Garbayo E., Imbuluzqueta I., Díaz-Herráez P., Abizanda G., Gavira J.J., Simón-Yarza T., Albiasu E., Tamayo E. (2014). Controlled Delivery of Fibroblast Growth Factor-1 and Neuregulin-1 from Biodegradable Microparticles Promotes Cardiac Repair in a Rat Myocardial Infarction Model through Activation of Endogenous Regeneration. J. Control. Release.

[115] Hubert F., Payan S.M., Rochais F. (2018). FGF10 Signaling in Heart Development, Homeostasis, Disease and Repair. Front. Genet..

[116] Rochais F., Sturny R., Chao C.-M., Mesbah K., Bennett M., Mohun T.J., Bellusci S., Kelly R.G. (2014). FGF10 Promotes Regional Foetal Cardiomyocyte Proliferation and Adult Cardiomyocyte Cell-Cycle Re-Entry. Cardiovasc. Res..

[117] Hubert F., Payan S.M., Pelce E., Bouchard L., Sturny R., Lenfant N., Mottola G., Collart F., Kelly R.G., Rochais F. (2022). FGF10 Promotes Cardiac Repair through a Dual Cellular Mechanism Increasing Cardiomyocyte Renewal and Inhibiting Fibrosis. Cardiovasc. Res..

[118] Díaz del Moral S., Benaouicha M., Muñoz-Chápuli R., Carmona R. (2021). The Insulin-like Growth Factor Signalling Pathway in Cardiac Development and Regeneration. Int. J. Mol. Sci..

[119] Huang Y., Harrison M.R., Osorio A., Kim J., Baugh A., Duan C., Sucov H.M., Lien C.-L. (2013). Igf Signaling Is Required for Cardiomyocyte Proliferation during Zebrafish Heart Development and Regeneration. PLoS ONE.

[120] Brown G.S., Jang J., Li D. (2023). Growth Factors and Their Roles in Cardiac Development and Regeneration: A Narrative Review. Pediatr. Med..

[121] Sui Y., Zhang W., Tang T., Gao L., Cao T., Zhu H., You Q., Yu B., Yang T. (2020). Insulin-like Growth Factor-II Overexpression Accelerates Parthenogenetic Stem Cell Differentiation into Cardiomyocytes and Improves Cardiac Function after Acute Myocardial Infarction in Mice. Stem Cell Res. Ther..

[122] Wang W., Yu K., Zhao S.-Y., Mo D.-G., Liu J.-H., Han L.-J., Li T., Yao H.-C. (2023). The Impact of Circulating IGF-1 and IGFBP-2 on Cardiovascular Prognosis in Patients with Acute Coronary Syndrome. Front. Cardiovasc. Med..

[123] Haywood N.J., Slater T.A., Drozd M., Warmke N., Matthews C., Cordell P.A., Smith J., Rainford J., Cheema H., Maher C. (2020). IGFBP-1 in Cardiometabolic Pathophysiology—Insights from Loss-of-Function and Gain-of-Function Studies in Male Mice. J. Endocr. Soc..

[124] Tang X., Jiang H., Lin P., Zhang Z., Chen M., Zhang Y., Mo J., Zhu Y., Liu N., Chen X. (2021). Insulin-like Growth Factor Binding Protein-1 Regulates HIF-1α Degradation to Inhibit Apoptosis in Hypoxic Cardiomyocytes. Cell Death Discov..

[125] Li S., Shen S., Xu H., Cai S., Yuan X., Wang C., Zhang X., Chen S., Chen J., Shi D.-L. (2023). IGF2BP3 Promotes Adult Myocardial Regeneration by Stabilizing MMP3 MRNA through Interaction with M6A Modification. Cell Death Discov..

[126] Farache Trajano L., Smart N. (2021). Immunomodulation for Optimal Cardiac Regeneration: Insights from Comparative Analyses. NPJ Regen. Med..

[127] Ponnusamy M., Li P.-F., Wang K. (2017). Understanding Cardiomyocyte Proliferation: An Insight into Cell Cycle Activity. Cell. Mol. Life Sci..

[128] Ikenishi A., Okayama H., Iwamoto N., Yoshitome S., Tane S., Nakamura K., Obayashi T., Hayashi T., Takeuchi T. (2012). Cell Cycle Regulation in Mouse Heart during Embryonic and Postnatal Stages. Dev. Growth Differ..

[129] Yücel D., Garay B.I., Perlingeiro R.C.R., van Berlo J.H. (2022). Stimulation of Cardiomyocyte Proliferation Is Dependent on Species and Level of Maturation. Front. Cell Dev. Biol..

[130] Yan Y., Miao D., Yang Z., Zhang D. (2019). Loss of P27 ^kip1^ Suppresses the Myocardial Senescence Caused by Estrogen Deficiency. J. Cell. Biochem..

[131] Di Stefano V., Giacca M., Capogrossi M.C., Crescenzi M., Martelli F. (2011). Knockdown of Cyclin-Dependent Kinase Inhibitors Induces Cardiomyocyte Re-Entry in the Cell Cycle. J. Biol. Chem..

[132] Tane S., Kubota M., Okayama H., Ikenishi A., Yoshitome S., Iwamoto N., Satoh Y., Kusakabe A., Ogawa S., Kanai A. (2014). Repression of Cyclin D1 Expression Is Necessary for the Maintenance of Cell Cycle Exit in Adult Mammalian Cardiomyocytes. J. Biol. Chem..

[133] Zhen L., Zhao Q., Lü J., Deng S., Xu Z., Zhang L., Zhang Y., Fan H., Chen X., Liu Z. (2020). MiR-301a-PTEN-AKT Signaling Induces Cardiomyocyte Proliferation and Promotes Cardiac Repair Post-MI. Mol. Ther. Nucleic Acids.

[134] Łukasik P., Załuski M., Gutowska I. (2021). Cyclin-Dependent Kinases (CDK) and Their Role in Diseases Development—Review. Int. J. Mol. Sci..

[135] Mohamed T.M.A., Ang Y.S., Radzinsky E., Zhou P., Huang Y., Elfenbein A., Foley A., Magnitsky S., Srivastava D. (2018). Regulation of Cell Cycle to Stimulate Adult Cardiomyocyte Proliferation and Cardiac Regeneration. Cell.

[136] Sun J., Wang L., Matthews R.C., Walcott G.P., Lu Y.-A., Wei Y., Zhou Y., Zangi L., Zhang J. (2023). CCND2 Modified MRNA Activates Cell Cycle of Cardiomyocytes in Hearts with Myocardial Infarction in Mice and Pigs. Circ. Res..

[137] Vujic A., Natarajan N., Lee R.T. (2020). Molecular Mechanisms of Heart Regeneration. Semin. Cell Dev. Biol..

[138] Hille S., Dierck F., Kühl C., Sosna J., Adam-Klages S., Adam D., Lüllmann-Rauch R., Frey N., Kuhn C. (2016). Dyrk1a Regulates the Cardiomyocyte Cell Cycle via D-Cyclin-Dependent Rb/E2f-Signalling. Cardiovasc. Res..

[139] Young A., Bradley L.A., Farrar E., Bilcheck H.O., Tkachenko S., Saucerman J.J., Bekiranov S., Wolf M.J. (2022). Inhibition of DYRK1a Enhances Cardiomyocyte Cycling After Myocardial Infarction. Circ. Res..

[140] Gong R., Gao X., Liu Y., Shen Y., Jiang Z., Wang X., Zagidullin N., Ma W., Wang N., Cai B. (2023). Cyclin L1 Controls Cardiomyocyte Proliferation and Heart Repair after Injury. Signal Transduct. Target. Ther..

[141] Loyer P., Trembley J.H. (2020). Roles of CDK/Cyclin Complexes in Transcription and Pre-MRNA Splicing: Cyclins L and CDK11 at the Cross-Roads of Cell Cycle and Regulation of Gene Expression. Semin. Cell Dev. Biol..

[142] Li Y., Hu S., Ma G., Yao Y., Yan G., Chen J., Li Y., Zhang Z. (2013). Acute Myocardial Infarction Induced Functional Cardiomyocytes to Re-Enter the Cell Cycle. Am. J. Transl. Res..

[143] Shapiro S.D., Ranjan A.K., Kawase Y., Cheng R.K., Kara R.J., Bhattacharya R., Guzman-Martinez G., Sanz J., Garcia M.J., Chaudhry H.W. (2014). Cyclin A2 Induces Cardiac Regeneration After Myocardial Infarction Through Cytokinesis of Adult Cardiomyocytes. Sci. Transl. Med..

[144] Huang S., Li X., Zheng H., Si X., Li B., Wei G., Li C., Chen Y., Chen Y., Liao W. (2019). Loss of Super-Enhancer-Regulated CircRNA Nfix Induces Cardiac Regeneration after Myocardial Infarction in Adult Mice. Circulation.

[145] Wang H., Yu W., Wang Y., Wu R., Dai Y., Deng Y., Wang S., Yuan J., Tan R. (2023). P53 Contributes to Cardiovascular Diseases via Mitochondria Dysfunction: A New Paradigm. Free Radic. Biol. Med..

[146] Mak T.W., Hauck L., Grothe D., Billia F. (2017). P53 Regulates the Cardiac Transcriptome. Proc. Natl. Acad. Sci. USA.

[147] Xiao Q., Zhang G., Wang H., Chen L., Lu S., Pan D., Liu G., Yang Z. (2017). A P53-Based Genetic Tracing System to Follow Postnatal Cardiomyocyte Expansion in Heart Regeneration. Development.

[148] Men H., Cai H., Cheng Q., Zhou W., Wang X., Huang S., Zheng Y., Cai L. (2021). The Regulatory Roles of P53 in Cardiovascular Health and Disease. Cell. Mol. Life Sci..

[149] Stanley-Hasnain S., Hauck L., Grothe D., Aschar-Sobbi R., Beca S., Butany J., Backx P.H., Mak T.W., Billia F. (2017). P53 and Mdm2 Act Synergistically to Maintain Cardiac Homeostasis and Mediate Cardiomyocyte Cell Cycle Arrest through a Network of MicroRNAs. Cell Cycle.

[150] Liu C.-Y., Zhang Y.-H., Li R.-B., Zhou L.-Y., An T., Zhang R.-C., Zhai M., Huang Y., Yan K.-W., Dong Y.-H. (2018). LncRNA CAIF Inhibits Autophagy and Attenuates Myocardial Infarction by Blocking P53-Mediated Myocardin Transcription. Nat. Commun..

[151] Padula S.L., Velayutham N., Yutzey K.E. (2021). Transcriptional Regulation of Postnatal Cardiomyocyte Maturation and Regeneration. Int. J. Mol. Sci..

[152] Secco I., Giacca M. (2023). Regulation of Endogenous Cardiomyocyte Proliferation: The Known Unknowns. J. Mol. Cell. Cardiol..

[153] Chakraborty S., Yutzey K.E. (2012). Tbx20 Regulation of Cardiac Cell Proliferation and Lineage Specialization during Embryonic and Fetal Development in Vivo. Dev. Biol..

[154] Chakraborty S., Sengupta A., Yutzey K.E. (2013). Tbx20 Promotes Cardiomyocyte Proliferation and Persistence of Fetal Characteristics in Adult Mouse Hearts. J. Mol. Cell. Cardiol..

[155] Xiang F., Guo M., Yutzey K.E. (2016). Overexpression of Tbx20 in Adult Cardiomyocytes Promotes Proliferation and Improves Cardiac Function After Myocardial Infarction. Circulation.

[156] Boogerd C.J., Zhu X., Aneas I., Sakabe N., Zhang L., Sobreira D.R., Montefiori L., Bogomolovas J., Joslin A.C., Zhou B. (2018). *Tbx20* Is Required in Mid-Gestation Cardiomyocytes and Plays a Central Role in Atrial Development. Circ. Res..

[157] Tang Y., Aryal S., Geng X., Zhou X., Fast V.G., Zhang J., Lu R., Zhou Y. (2022). TBX20 Improves Contractility and Mitochondrial Function During Direct Human Cardiac Reprogramming. Circulation.

[158] Hausenloy D.J., Chilian W., Crea F., Davidson S.M., Ferdinandy P., Garcia-Dorado D., van Royen N., Schulz R., Heusch G. (2019). The Coronary Circulation in Acute Myocardial Ischaemia/Reperfusion Injury: A Target for Cardioprotection. Cardiovasc. Res..

[159] He X., Du T., Long T., Liao X., Dong Y., Huang Z.-P. (2022). Signaling Cascades in the Failing Heart and Emerging Therapeutic Strategies. Signal Transduct. Target. Ther..

[160] Long H., Steimle J.D., Grisanti Canozo F.J., Kim J.H., Li X., Morikawa Y., Park M., Turaga D., Adachi I., Wythe J.D. (2024). Endothelial Cells Adopt a Pro-Reparative Immune Responsive Signature during Cardiac Injury. Life Sci. Alliance.

[161] Berkeley B., Tang M.N.H., Brittan M. (2023). Mechanisms Regulating Vascular and Lymphatic Regeneration in the Heart after Myocardial Infarction. J. Pathol..

[162] Tombor L.S., John D., Glaser S.F., Luxán G., Forte E., Furtado M., Rosenthal N., Baumgarten N., Schulz M.H., Wittig J. (2021). Single Cell Sequencing Reveals Endothelial Plasticity with Transient Mesenchymal Activation after Myocardial Infarction. Nat. Commun..

[163] He L., Huang X., Kanisicak O., Li Y., Wang Y., Li Y., Pu W., Liu Q., Zhang H., Tian X. (2017). Preexisting Endothelial Cells Mediate Cardiac Neovascularization after Injury. J. Clin. Investig..

[164] Wu X., Reboll M.R., Korf-Klingebiel M., Wollert K.C. (2021). Angiogenesis after Acute Myocardial Infarction. Cardiovasc. Res..

[165] Shi W., Xin Q., Yuan R., Yuan Y., Cong W., Chen K. (2021). Neovascularization: The Main Mechanism of MSCs in Ischemic Heart Disease Therapy. Front. Cardiovasc. Med..

[166] Malektaj H., Nour S., Imani R., Siadati M.H. (2023). Angiogenesis Induction as a Key Step in Cardiac Tissue Regeneration: From Angiogenic Agents to Biomaterials. Int. J. Pharm..

[167] Tian X., Zhou B. (2022). Coronary Vessel Formation in Development and Regeneration: Origins and Mechanisms. J. Mol. Cell. Cardiol..

[168] Du Y., Ge Y., Xu Z., Aa N., Gu X., Meng H., Lin Z., Zhu D., Shi J., Zhuang R. (2018). Hypoxia-Inducible Factor 1 Alpha (HIF-1α)/Vascular Endothelial Growth Factor (VEGF) Pathway Participates in Angiogenesis of Myocardial Infarction in Muscone-Treated Mice: Preliminary Study. Med. Sci. Monit..

[169] Shi X.-Q., Chen G., Tan J.-Q., Li Z., Chen S.-M., He J.-H., Zhang L., Xu H.-X. (2022). Total Alkaloid Fraction of Leonurus Japonicus Houtt. Promotes Angiogenesis and Wound Healing through SRC/MEK/ERK Signaling Pathway. J. Ethnopharmacol..

[170] Chen M.H., Fu Q.M., Yang Z. (2020). The Roles of AMPK in Revascularization. Cardiol. Res. Pract..

[171] Yetkin-Arik B., Vogels I.M.C., Neyazi N., van Duinen V., Houtkooper R.H., van Noorden C.J.F., Klaassen I., Schlingemann R.O. (2019). Endothelial Tip Cells in Vitro Are Less Glycolytic and Have a More Flexible Response to Metabolic Stress than Non-Tip Cells. Sci. Rep..

[172] Dittrich G.M., Froese N., Wang X., Kroeger H., Wang H., Szaroszyk M., Malek-Mohammadi M., Cordero J., Keles M., Korf-Klingebiel M. (2021). Fibroblast GATA-4 and GATA-6 Promote Myocardial Adaptation to Pressure Overload by Enhancing Cardiac Angiogenesis. Basic Res. Cardiol..

[173] Sturny R., Boulgakoff L., Kelly R.G., Miquerol L. (2024). Transient Formation of Collaterals Contributes to the Restoration of the Arterial Tree during Cardiac Regeneration in Neonatal Mice. J. Mol. Cell. Cardiol..

[174] van Royen N., Piek J.J., Schaper W., Fulton W.F. (2009). A Critical Review of Clinical Arteriogenesis Research. J. Am. Coll. Cardiol..

[175] Cochain C., Zernecke A. (2015). Stimulating Arteriogenesis but Not Atherosclerosis: IFN-α/β Receptor Subunit 1 as a Novel Therapeutic Target: Figure 1. Cardiovasc. Res..

[176] Spadaccio C., Nenna A., Rose D., Piccirillo F., Nusca A., Grigioni F., Chello M., Vlahakes G.J. (2022). The Role of Angiogenesis and Arteriogenesisin Myocardial Infarction and Coronary Revascularization. J. Cardiovasc. Transl. Res..

[177] Ribatti D., Vacca A., Nico B., Roncali L., Dammacco F. (2001). Postnatal Vasculogenesis. Mech. Dev..

[178] Martín-Bórnez M., Falcón D., Morrugares R., Siegfried G., Khatib A.-M., Rosado J.A., Galeano-Otero I., Smani T. (2023). New Insights into the Reparative Angiogenesis after Myocardial Infarction. Int. J. Mol. Sci..

[179] Gong H., Wang T., Xu Q. (2021). Resident Stem Cells in the Heart. Med. Rev..

[180] Xing S., Tian J.Z., Yang S.H., Huang X.T., Ding Y.F., Lu Q.Y., Yang J.S., Yang W.J. (2021). Setd4 Controlled Quiescent C-Kit^+^ Cells Contribute to Cardiac Neovascularization of Capillaries beyond Activation. Sci. Rep..

[181] Pelliccia F., Zimarino M., De Luca G., Viceconte N., Tanzilli G., De Caterina R. (2022). Endothelial Progenitor Cells in Coronary Artery Disease: From Bench to Bedside. Stem Cells Transl. Med..

[182] Johnson T., Zhao L., Manuel G., Taylor H., Liu D. (2019). Approaches to Therapeutic Angiogenesis for Ischemic Heart Disease. J. Mol. Med..

[183] Huang H., Huang W. (2022). Regulation of Endothelial Progenitor Cell Functions in Ischemic Heart Disease: New Therapeutic Targets for Cardiac Remodeling and Repair. Front. Cardiovasc. Med..

[184] Asahara T., Murohara T., Sullivan A., Silver M., van der Zee R., Li T., Witzenbichler B., Schatteman G., Isner J.M. (1997). Isolation of Putative Progenitor Endothelial Cells for Angiogenesis. Science.

[185] Marvasti T.B., Alibhai F.J., Weisel R.D., Li R.-K. (2019). CD34^+^ Stem Cells: Promising Roles in Cardiac Repair and Regeneration. Can. J. Cardiol..

[186] Hassanpour M., Salybekov A.A., Kobayashi S., Asahara T. (2023). CD34 Positive Cells as Endothelial Progenitor Cells in Biology and Medicine. Front. Cell Dev. Biol..

[187] Ingram D.A., Mead L.E., Moore D.B., Woodard W., Fenoglio A., Yoder M.C. (2005). Vessel Wall–Derived Endothelial Cells Rapidly Proliferate Because They Contain a Complete Hierarchy of Endothelial Progenitor Cells. Blood.

[188] Asahara T., Masuda H., Takahashi T., Kalka C., Pastore C., Silver M., Kearne M., Magner M., Isner J.M. (1999). Bone Marrow Origin of Endothelial Progenitor Cells Responsible for Postnatal Vasculogenesis in Physiological and Pathological Neovascularization. Circ. Res..

[189] Hueso L., Rios-Navarro C., Ruiz-Sauri A., Chorro F.J., Nunez J., Sanz M.J., Bodi V., Piqueras L. (2017). Dynamics and Implications of Circulating Anti-Angiogenic VEGF-A165b Isoform in Patients with ST-Elevation Myocardial Infarction. Sci. Rep..

[190] Sabbah N., Tamari T., Elimelech R., Doppelt O., Rudich U., Zigdon-Giladi H. (2019). Predicting Angiogenesis by Endothelial Progenitor Cells Relying on In-Vitro Function Assays and VEGFR-2 Expression Levels. Biomolecules.

[191] Yellowley C.E., Toupadakis C.A., Vapniarsky N., Wong A. (2019). Circulating Progenitor Cells and the Expression of Cxcl12, Cxcr4 and Angiopoietin-like 4 during Wound Healing in the Murine Ear. PLoS ONE.

[192] Short W.D., Steen E., Kaul A., Wang X., Olutoye O.O., Vangapandu H.V., Templeman N., Blum A.J., Moles C.M., Narmoneva D.A. (2022). IL-10 Promotes Endothelial Progenitor Cell Infiltration and Wound Healing via STAT3. The FASEB Journal.

[193] Yue Y., Wang C., Benedict C., Huang G., Truongcao M., Roy R., Cimini M., Garikipati V.N.S., Cheng Z., Koch W.J. (2020). Interleukin-10 Deficiency Alters Endothelial Progenitor Cell–Derived Exosome Reparative Effect on Myocardial Repair via Integrin-Linked Kinase Enrichment. Circ. Res..

[194] Zeng C.-Y., Xu J., Liu X., Lu Y.-Q. (2021). Cardioprotective Roles of Endothelial Progenitor Cell-Derived Exosomes. Front. Cardiovasc. Med..

[195] Huang H., Xu Z., Qi Y., Zhang W., Zhang C., Jiang M., Deng S., Wang H. (2020). Exosomes from SIRT1-Overexpressing ADSCs Restore Cardiac Function by Improving Angiogenic Function of EPCs. Mol. Ther. Nucleic Acids.

[196] Rehman J., Li J., Orschell C.M., March K.L. (2003). Peripheral Blood “Endothelial Progenitor Cells” Are Derived from Monocyte/Macrophages and Secrete Angiogenic Growth Factors. Circulation.

[197] Deutsch M.-A., Brunner S., Grabmaier U., David R., Ott I., Huber B.C. (2020). Cardioprotective Potential of Human Endothelial-Colony Forming Cells from Diabetic and Nondiabetic Donors. Cells.

[198] Medina R.J., Barber C.L., Sabatier F., Dignat-George F., Melero-Martin J.M., Khosrotehrani K., Ohneda O., Randi A.M., Chan J.K.Y., Yamaguchi T. (2017). Endothelial Progenitors: A Consensus Statement on Nomenclature. Stem Cells Transl. Med..

[199] Banno K., Yoder M.C. (2018). Tissue Regeneration Using Endothelial Colony-Forming Cells: Promising Cells for Vascular Repair. Pediatr. Res..

[200] Popescu S., Preda M.B., Marinescu C.I., Simionescu M., Burlacu A. (2021). Dual Stem Cell Therapy Improves the Myocardial Recovery Post-Infarction through Reciprocal Modulation of Cell Functions. Int. J. Mol. Sci..

[201] Fujisawa T., Tura-Ceide O., Hunter A., Mitchell A., Vesey A., Medine C., Gallogly S., Hadoke P.W.F., Keith C., Sproul A. (2019). Endothelial Progenitor Cells Do Not Originate from the Bone Marrow. Circulation.

[202] Li Z., Solomonidis E.G., Meloni M., Taylor R.S., Duffin R., Dobie R., Magalhaes M.S., Henderson B.E.P., Louwe P.A., D’Amico G. (2019). Single-Cell Transcriptome Analyses Reveal Novel Targets Modulating Cardiac Neovascularization by Resident Endothelial Cells Following Myocardial Infarction. Eur. Heart J..

[203] Abdelgawad M.E., Desterke C., Uzan G., Naserian S. (2021). Single-Cell Transcriptomic Profiling and Characterization of Endothelial Progenitor Cells: New Approach for Finding Novel Markers. Stem Cell Res. Ther..

[204] (2022). Endothelial Progenitor Cell-Derived Small Extracellular Vesicles for Myocardial Angiogenesis and Revascularization. J. Clin. Transl. Res..

[205] Chen C.W., Wang L.L., Zaman S., Gordon J., Arisi M.F., Venkataraman C.M., Chung J.J., Hung G., Gaffey A.C., Spruce L.A. (2018). Sustained Release of Endothelial Progenitor Cell-Derived Extracellular Vesicles from Shear-Thinning Hydrogels Improves Angiogenesis and Promotes Function after Myocardial Infarction. Cardiovasc. Res..

[206] Dergilev K., Tsokolaeva Z., Makarevich P., Beloglazova I., Zubkova E., Boldyreva M., Ratner E., Dyikanov D., Menshikov M., Ovchinnikov A. (2018). C-Kit Cardiac Progenitor Cell Based Cell Sheet Improves Vascularization and Attenuates Cardiac Remodeling Following Myocardial Infarction in Rats. Biomed Res. Int..

[207] Roefs M.T., Bauzá-Martinez J., van de Wakker S.I., Qin J., Olijve W.T., Tuinte R., Rozeboom M., Snijders Blok C., Mol E.A., Wu W. (2023). Cardiac Progenitor Cell-Derived Extracellular Vesicles Promote Angiogenesis through Both Associated- and Co-Isolated Proteins. Commun. Biol..

[208] Ceja L., Escopete S.S., Hughes L., Lopez L.V., Camberos V., Vallejos P., Wall N.R., Kearns-Jonker M. (2023). Neonatal Cardiovascular-Progenitor-Cell-Derived Extracellular Vesicles Activate YAP1 in Adult Cardiac Progenitor Cells. Int. J. Mol. Sci..

[209] Vrijsen K.R., Maring J.A., Chamuleau S.A.J., Verhage V., Mol E.A., Deddens J.C., Metz C.H.G., Lodder K., van Eeuwijk E.C.M., van Dommelen S.M. (2016). Exosomes from Cardiomyocyte Progenitor Cells and Mesenchymal Stem Cells Stimulate Angiogenesis Via EMMPRIN. Adv. Healthc. Mater..

[210] Barile L., Lionetti V., Cervio E., Matteucci M., Gherghiceanu M., Popescu L.M., Torre T., Siclari F., Moccetti T., Vassalli G. (2014). Extracellular Vesicles from Human Cardiac Progenitor Cells Inhibit Cardiomyocyte Apoptosis and Improve Cardiac Function after Myocardial Infarction. Cardiovasc. Res..

[211] Youn S.-W., Li Y., Kim Y.-M., Sudhahar V., Abdelsaid K., Kim H.W., Liu Y., Fulton D.J.R., Ashraf M., Tang Y. (2019). Modification of Cardiac Progenitor Cell-Derived Exosomes by MiR-322 Provides Protection against Myocardial Infarction through Nox2-Dependent Angiogenesis. Antioxidants.

[212] Poomani M.S., Mariappan I., Perumal R., Regurajan R., Muthan K., Subramanian V. (2022). Mesenchymal Stem Cell (MSCs) Therapy for Ischemic Heart Disease: A Promising Frontier. Glob. Heart.

[213] Wagner M.J., Khan M., Mohsin S. (2020). Healing the Broken Heart; The Immunomodulatory Effects of Stem Cell Therapy. Front. Immunol..

[214] Tran T., Cruz C., Chan A., Awad S., Rajasingh J., Deth R., Gurusamy N. (2023). Mesenchymal Stem Cell-Derived Long Noncoding RNAs in Cardiac Injury and Repair. Cells.

[215] Klopsch C., Skorska A., Ludwig M., Gaebel R., Lemcke H., Kleiner G., Beyer M., Vollmar B., David R., Steinhoff G. (2017). Cardiac Mesenchymal Stem Cells Proliferate Early in the Ischemic Heart. Eur. Surg. Res..

[216] Klopsch C., Skorska A., Ludwig M., Lemcke H., Maass G., Gaebel R., Beyer M., Lux C., Toelk A., Müller K. (2018). Intramyocardial Angiogenetic Stem Cells and Epicardial Erythropoietin Save the Acute Ischemic Heart. Dis. Model Mech..

[217] Gong X., Liu H., Wang S., Liang S., Wang G. (2019). Exosomes Derived from SDF1-overexpressing Mesenchymal Stem Cells Inhibit Ischemic Myocardial Cell Apoptosis and Promote Cardiac Endothelial Microvascular Regeneration in Mice with Myocardial Infarction. J. Cell. Physiol..

[218] Qian Z., Sharma D., Jia W., Radke D., Kamp T., Zhao F. (2019). Engineering Stem Cell Cardiac Patch with Microvascular Features Representative of Native Myocardium. Theranostics.

[219] Välimäki M.J., Leigh R.S., Kinnunen S.M., March A.R., de Sande A.H., Kinnunen M., Varjosalo M., Heinäniemi M., Kaynak B.L., Ruskoaho H. (2021). GATA-Targeted Compounds Modulate Cardiac Subtype Cell Differentiation in Dual Reporter Stem Cell Line. Stem Cell Res. Ther..

[220] He J.G., Li H.R., Li B.B., Xie Q.L., Yan D., Wang X.J. (2019). Bone Marrow Mesenchymal Stem Cells Overexpressing GATA-4 Improve Cardiac Function Following Myocardial Infarction. Perfusion.

[221] Gong M., Wang M., Xu J., Yu B., Wang Y.-G., Liu M., Ashraf M., Xu M. (2022). Nano-Sized Extracellular Vesicles Secreted from GATA-4 Modified Mesenchymal Stem Cells Promote Angiogenesis by Delivering Let-7 MiRNAs. Cells.

[222] He J.-G., Li H.-R., Han J.-X., Li B.-B., Yan D., Li H.-Y., Wang P., Luo Y. (2018). GATA-4-Expressing Mouse Bone Marrow Mesenchymal Stem Cells Improve Cardiac Function after Myocardial Infarction via Secreted Exosomes. Sci. Rep..

[223] Mia M.M., Singh M.K. (2019). The Hippo Signaling Pathway in Cardiac Development and Diseases. Front. Cell Dev. Biol..

[224] Zhong Z., Jiao Z., Yu F.-X. (2024). The Hippo Signaling Pathway in Development and Regeneration. Cell. Rep..

[225] Bornhorst D., Abdelilah-Seyfried S. (2021). Strong as a Hippo’s Heart: Biomechanical Hippo Signaling During Zebrafish Cardiac Development. Front. Cell Dev. Biol..

[226] Wang Y., Yu A., Yu F.-X. (2017). The Hippo Pathway in Tissue Homeostasis and Regeneration. Protein Cell.

[227] Morikawa Y., Zhang M., Heallen T., Leach J., Tao G., Xiao Y., Bai Y., Li W., Willerson J.T., Martin J.F. (2015). Actin Cytoskeletal Remodeling with Protrusion Formation Is Essential for Heart Regeneration in Hippo-Deficient Mice. Sci. Signal.

[228] Xin M., Kim Y., Sutherland L.B., Murakami M., Qi X., McAnally J., Porrello E.R., Mahmoud A.I., Tan W., Shelton J.M. (2013). Hippo Pathway Effector Yap Promotes Cardiac Regeneration. Proc. Natl. Acad. Sci. USA.

[229] Del Re D.P., Yang Y., Nakano N., Cho J., Zhai P., Yamamoto T., Zhang N., Yabuta N., Nojima H., Pan D. (2013). Yes-Associated Protein Isoform 1 (Yap1) Promotes Cardiomyocyte Survival and Growth to Protect against Myocardial Ischemic Injury. J. Biol. Chem..

[230] Heallen T., Morikawa Y., Leach J., Tao G., Willerson J.T., Johnson R.L., Martin J.F. (2013). Hippo Signaling Impedes Adult Heart Regeneration. Development.

[231] Leach J.P., Heallen T., Zhang M., Rahmani M., Morikawa Y., Hill M.C., Segura A., Willerson J.T., Martin J.F. (2017). Hippo Pathway Deficiency Reverses Systolic Heart Failure after Infarction. Nature.

[232] Tao G., Kahr P.C., Morikawa Y., Zhang M., Rahmani M., Heallen T.R., Li L., Sun Z., Olson E.N., Amendt B.A. (2016). Pitx2 Promotes Heart Repair by Activating the Antioxidant Response after Cardiac Injury. Nature.

[233] Li H., Chang C., Li X., Zhang R. (2021). The Roles and Activation of Endocardial Notch Signaling in Heart Regeneration. Cell Regeneration.

[234] Sachan N., Sharma V., Mutsuddi M., Mukherjee A. (2024). Notch Signalling: Multifaceted Role in Development and Disease. FEBS J..

[235] Mack J.J., Mosqueiro T.S., Archer B.J., Jones W.M., Sunshine H., Faas G.C., Briot A., Aragón R.L., Su T., Romay M.C. (2017). NOTCH1 Is a Mechanosensor in Adult Arteries. Nat. Commun..

[236] Pitulescu M.E., Schmidt I., Giaimo B.D., Antoine T., Berkenfeld F., Ferrante F., Park H., Ehling M., Biljes D., Rocha S.F. (2017). Dll4 and Notch Signalling Couples Sprouting Angiogenesis and Artery Formation. Nat. Cell Biol..

[237] Zou J., Fei Q., Xiao H., Wang H., Liu K., Liu M., Zhang H., Xiao X., Wang K., Wang N. (2019). VEGF-A Promotes Angiogenesis after Acute Myocardial Infarction through Increasing ROS Production and Enhancing ER Stress-mediated Autophagy. J. Cell. Physiol..

[238] Wang K., Ding R., Ha Y., Jia Y., Liao X., Wang S., Li R., Shen Z., Xiong H., Guo J. (2018). Hypoxia-Stressed Cardiomyocytes Promote Early Cardiac Differentiation of Cardiac Stem Cells through HIF-1α/Jagged1/Notch1 Signaling. Acta Pharm. Sin. B.

[239] Zhou X., Zhu R., Liu S., Xu H., Xu X., Wu Q., Liu J. (2018). Notch Signaling Promotes Angiogenesis and Improves Cardiac Function after Myocardial Infarction. J. Cell. Biochem..

[240] Xuan W., Khan M., Ashraf M. (2020). Extracellular Vesicles from Notch Activated Cardiac Mesenchymal Stem Cells Promote Myocyte Proliferation and Neovasculogenesis. Front. Cell Dev. Biol..

[241] Gude N.A., Emmanuel G., Wu W., Cottage C.T., Fischer K., Quijada P., Muraski J.A., Alvarez R., Rubio M., Schaefer E. (2008). Activation of Notch-Mediated Protective Signaling in the Myocardium. Circ. Res..

[242] Sciarretta S., Forte M., Frati G., Sadoshima J. (2018). New Insights into the Role of MTOR Signaling in the Cardiovascular System. Circ. Res..

[243] Apte R.S., Chen D.S., Ferrara N. (2019). VEGF in Signaling and Disease: Beyond Discovery and Development. Cell.

[244] Zhao L., Borikova A.L., Ben-Yair R., Guner-Ataman B., MacRae C.A., Lee R.T., Burns C.G., Burns C.E. (2014). Notch Signaling Regulates Cardiomyocyte Proliferation during Zebrafish Heart Regeneration. Proc. Natl. Acad. Sci. USA.

[245] Wang T., Chen X., Wang K., Ju J., Yu X., Yu W., Liu C., Wang Y. (2024). Cardiac Regeneration: Pre-Existing Cardiomyocyte as the Hub of Novel Signaling Pathway. Genes Dis..

[246] Croquelois A., Domenighetti A.A., Nemir M., Lepore M., Rosenblatt-Velin N., Radtke F., Pedrazzini T. (2008). Control of the Adaptive Response of the Heart to Stress via the Notch1 Receptor Pathway. J. Exp. Med..

[247] Felician G., Collesi C., Lusic M., Martinelli V., Ferro M.D., Zentilin L., Zacchigna S., Giacca M. (2014). Epigenetic Modification at Notch Responsive Promoters Blunts Efficacy of Inducing Notch Pathway Reactivation After Myocardial Infarction. Circ. Res..

[248] Nemir M., Metrich M., Plaisance I., Lepore M., Cruchet S., Berthonneche C., Sarre A., Radtke F., Pedrazzini T. (2014). The Notch Pathway Controls Fibrotic and Regenerative Repair in the Adult Heart. Eur. Heart J..

[249] Perveen S., Vanni R., Lo Iacono M., Rastaldo R., Giachino C. (2023). Direct Reprogramming of Resident Non-Myocyte Cells and Its Potential for In Vivo Cardiac Regeneration. Cells.

[250] He Y., Pang S., Huang J., Zhu K., Tong J., Tang Y., Ma G., Chen L. (2018). Blockade of RBP-J-Mediated Notch Signaling Pathway Exacerbates Cardiac Remodeling after Infarction by Increasing Apoptosis in Mice. Biomed Res. Int..

[251] Liu J., Xiao Q., Xiao J., Niu C., Li Y., Zhang X., Zhou Z., Shu G., Yin G. (2022). Wnt/β-Catenin Signalling: Function, Biological Mechanisms, and Therapeutic Opportunities. Signal Transduct. Target. Ther..

[252] Ni B., Sun M., Zhao J., Wang J., Cao Z. (2023). The Role of β-Catenin in Cardiac Diseases. Front. Pharmacol..

[253] Matteucci M., Casieri V., Gabisonia K., Aquaro G.D., Agostini S., Pollio G., Diamanti D., Rossi M., Travagli M., Porcari V. (2016). Magnetic Resonance Imaging of Infarct-Induced Canonical Wingless/Integrated (Wnt)/β-Catenin/T-Cell Factor Pathway Activation, in Vivo. Cardiovasc. Res..

[254] Aisagbonhi O., Rai M., Ryzhov S., Atria N., Feoktistov I., Hatzopoulos A.K. (2011). Experimental Myocardial Infarction Triggers Canonical Wnt Signaling and Endothelial-to-Mesenchymal Transition. Dis. Model Mech..

[255] Fan Y., Ho B.X., Pang J.K.S., Pek N.M.Q., Hor J.H., Ng S.-Y., Soh B.-S. (2018). Wnt/β-Catenin-Mediated Signaling Re-Activates Proliferation of Matured Cardiomyocytes. Stem Cell Res. Ther..

[256] Zelarayán L.C., Noack C., Sekkali B., Kmecova J., Gehrke C., Renger A., Zafiriou M.-P., van der Nagel R., Dietz R., de Windt L.J. (2008). β-Catenin Downregulation Attenuates Ischemic Cardiac Remodeling through Enhanced Resident Precursor Cell Differentiation. Proc. Natl. Acad. Sci. USA.

[257] Hodgkinson C.P., Gomez J.A., Baksh S.S., Payne A., Schmeckpeper J., Pratt R.E., Dzau V.J. (2018). Insights from Molecular Signature of in Vivo Cardiac C-Kit(+) Cells Following Cardiac Injury and β-Catenin Inhibition. J. Mol. Cell. Cardiol..

[258] Fu W., Wang W.E., Zeng C. (2019). Wnt Signaling Pathways in Myocardial Infarction and the Therapeutic Effects of Wnt Pathway Inhibitors. Acta Pharmacol. Sin..

[259] Baruah J., Hitzman R., Zhang J., Chaudhuri S., Mastej V., Wary K.K. (2017). The Allosteric Glycogen Synthase Kinase-3 Inhibitor NP12 Limits Myocardial Remodeling and Promotes Angiogenesis in an Acute Myocardial Infarction Model. J. Biol. Chem..

[260] Moheimani F., Roth H.M., Cross J., Reid A.T., Shaheen F., Warner S.M., Hirota J.A., Kicic A., Hallstrand T.S., Kahn M. (2015). Disruption of β-Catenin/CBP Signaling Inhibits Human Airway Epithelial–Mesenchymal Transition and Repair. Int. J. Biochem. Cell Biol..

[261] Paik D.T., Rai M., Ryzhov S., Sanders L.N., Aisagbonhi O., Funke M.J., Feoktistov I., Hatzopoulos A.K. (2015). Wnt10b Gain-of-Function Improves Cardiac Repair by Arteriole Formation and Attenuation of Fibrosis. Circ. Res..

[262] Mohri T., Iwakura T., Nakayama H., Fujio Y. (2012). JAK-STAT Signaling in Cardiomyogenesis of Cardiac Stem Cells. JAK-STAT.

[263] Hu X., Li J., Fu M., Zhao X., Wang W. (2021). The JAK/STAT Signaling Pathway: From Bench to Clinic. Signal Transduct. Target. Ther..

[264] Barry S.P., Townsend P.A., Latchman D.S., Stephanou A. (2007). Role of the JAK–STAT Pathway in Myocardial Injury. Trends Mol. Med..

[265] Zhu J., Yao K., Guo J., Shi H., Ma L., Wang Q., Liu H., Gao W., Sun A., Zou Y. (2017). MiR-181a and MiR-150 Regulate Dendritic Cell Immune Inflammatory Responses and Cardiomyocyte Apoptosis via Targeting JAK1–STAT1/c-Fos Pathway. J. Cell. Mol. Med..

[266] Boengler K., Hilfikerkleiner D., Drexler H., Heusch G., Schulz R. (2008). The Myocardial JAK/STAT Pathway: From Protection to Failure. Pharmacol. Ther..

[267] Zhao W., Chen Y., Yang W., Han Y., Wang Z., Huang F., Qiu Z., Yang K., Jin W. (2020). Effects of Cardiomyocyte-Specific Deletion of STAT3–A Murine Model of Heart Failure with Preserved Ejection Fraction. Front. Cardiovasc. Med..

[268] Wang N., Liu C., Wang X., He T., Li L., Liang X., Wang L., Song L., Wei Y., Wu Q. (2019). Hyaluronic Acid Oligosaccharides Improve Myocardial Function Reconstruction and Angiogenesis against Myocardial Infarction by Regulation of Macrophages. Theranostics.

[269] Zhang Q., Wang L., Wang S., Cheng H., Xu L., Pei G., Wang Y., Fu C., Jiang Y., He C. (2022). Signaling Pathways and Targeted Therapy for Myocardial Infarction. Signal Transduct. Target. Ther..

[270] Dunaeva M., Waltenberger J. (2017). Hh Signaling in Regeneration of the Ischemic Heart. Cell. Mol. Life Sci..

[271] Wang Y., Lu P., Zhao D., Sheng J. (2017). Targeting the Hedgehog Signaling Pathway for Cardiac Repair and Regeneration. Herz.

[272] Jing J., Wu Z., Wang J., Luo G., Lin H., Fan Y., Zhou C. (2023). Hedgehog Signaling in Tissue Homeostasis, Cancers and Targeted Therapies. Signal Transduct. Target. Ther..

[273] Kawagishi H., Xiong J., Rovira I.I., Pan H., Yan Y., Fleischmann B.K., Yamada M., Finkel T. (2018). Sonic Hedgehog Signaling Regulates the Mammalian Cardiac Regenerative Response. J. Mol. Cell. Cardiol..

[274] Gupta R., Mackie A.R., Misener S., Liu L., Losordo D.W., Kishore R. (2018). Endothelial Smoothened-Dependent Hedgehog Signaling Is Not Required for Sonic Hedgehog Induced Angiogenesis or Ischemic Tissue Repair. Lab. Investig..

[275] Giarretta I., Gaetani E., Bigossi M., Tondi P., Asahara T., Pola R. (2019). The Hedgehog Signaling Pathway in Ischemic Tissues. Int. J. Mol. Sci..

[276] Hanna A., Frangogiannis N.G. (2019). The Role of the TGF-β Superfamily in Myocardial Infarction. Front. Cardiovasc. Med..

[277] Frangogiannis N.G. (2017). The Role of Transforming Growth Factor (TGF)-β in the Infarcted Myocardium. J. Thorac. Dis..

[278] Chablais F., Veit J., Rainer G., Jaźwińska A. (2011). The Zebrafish Heart Regenerates after Cryoinjury-Induced Myocardial Infarction. BMC Dev. Biol..

[279] Peng Y., Wang W., Fang Y., Hu H., Chang N., Pang M., Hu Y.-F., Li X., Long H., Xiong J.-W. (2021). Inhibition of TGF-β/Smad3 Signaling Disrupts Cardiomyocyte Cell Cycle Progression and Epithelial–Mesenchymal Transition-Like Response During Ventricle Regeneration. Front. Cell Dev. Biol..

[280] Vanwijk B., Moorman A., Vandenhoff M. (2007). Role of Bone Morphogenetic Proteins in Cardiac Differentiation. Cardiovasc. Res..

[281] Katagiri T., Watabe T. (2016). Bone Morphogenetic Proteins. Cold Spring Harb. Perspect. Biol..

[282] Wu C.-C., Kruse F., Vasudevarao M.D., Junker J.P., Zebrowski D.C., Fischer K., Noël E.S., Grün D., Berezikov E., Engel F.B. (2016). Spatially Resolved Genome-Wide Transcriptional Profiling Identifies BMP Signaling as Essential Regulator of Zebrafish Cardiomyocyte Regeneration. Dev. Cell.

[283] Prados B., Gómez-Apiñániz P., Papoutsi T., Luxán G., Zaffran S., Pérez-Pomares J.M., de la Pompa J.L. (2018). Myocardial Bmp2 Gain Causes Ectopic EMT and Promotes Cardiomyocyte Proliferation and Immaturity. Cell Death Dis..

[284] Jiang H., Salmon R.M., Upton P.D., Wei Z., Lawera A., Davenport A.P., Morrell N.W., Li W. (2016). The Prodomain-Bound Form of Bone Morphogenetic Protein 10 Is Biologically Active on Endothelial Cells. J. Biol. Chem..

[285] Qu X., Liu Y., Cao D., Chen J., Liu Z., Ji H., Chen Y., Zhang W., Zhu P., Xiao D. (2019). BMP10 Preserves Cardiac Function through Its Dual Activation of SMAD-Mediated and STAT3-Mediated Pathways. J. Biol. Chem..

[286] Sun L., Yu J., Qi S., Hao Y., Liu Y., Li Z. (2014). Bone Morphogenetic Protein-10 Induces Cardiomyocyte Proliferation and Improves Cardiac Function after Myocardial Infarction. J. Cell. Biochem..

[287] Gil-Cabrerizo P., Scacchetti I., Garbayo E., Blanco-Prieto M.J. (2023). Cardiac Tissue Engineering for Myocardial Infarction Treatment. Eur. J. Pharm. Sci..

[288] Lam N.T., Sadek H.A. (2018). Neonatal Heart Regeneration. Circulation.

[289] Sacco A.M., Castaldo C., Di Meglio F., Nurzynska D., Palermi S., Spera R., Gnasso R., Zinno G., Romano V., Belviso I. (2023). The Long and Winding Road to Cardiac Regeneration. Appl. Sci..

[290] Yan W., Xia Y., Zhao H., Xu X., Ma X., Tao L. (2024). Stem Cell-Based Therapy in Cardiac Repair after Myocardial Infarction: Promise, Challenges, and Future Directions. J. Mol. Cell. Cardiol..

[291] Jackson K.A., Majka S.M., Wang H., Pocius J., Hartley C.J., Majesky M.W., Entman M.L., Michael L.H., Hirschi K.K., Goodell M.A. (2001). Regeneration of Ischemic Cardiac Muscle and Vascular Endothelium by Adult Stem Cells. J. Clin. Investig..

[292] Park S.-J., Kim R.Y., Park B.-W., Lee S., Choi S.W., Park J.-H., Choi J.J., Kim S.-W., Jang J., Cho D.-W. (2019). Dual Stem Cell Therapy Synergistically Improves Cardiac Function and Vascular Regeneration Following Myocardial Infarction. Nat. Commun..

[293] Nygren J.M., Jovinge S., Breitbach M., Säwén P., Röll W., Hescheler J., Taneera J., Fleischmann B.K., Jacobsen S.E.W. (2004). Bone Marrow–Derived Hematopoietic Cells Generate Cardiomyocytes at a Low Frequency through Cell Fusion, but Not Transdifferentiation. Nat. Med..

[294] Liew L.C., Ho B.X., Soh B.-S. (2020). Mending a Broken Heart: Current Strategies and Limitations of Cell-Based Therapy. Stem Cell Res. Ther..

[295] Lee H., Cho H.-J., Han Y., Lee S.H. (2024). Mid- to Long-Term Efficacy and Safety of Stem Cell Therapy for Acute Myocardial Infarction: A Systematic Review and Meta-Analysis. Stem Cell Res. Ther..

[296] Lyra-Leite D.M., Gutiérrez-Gutiérrez Ó., Wang M., Zhou Y., Cyganek L., Burridge P.W. (2022). A Review of Protocols for Human IPSC Culture, Cardiac Differentiation, Subtype-Specification, Maturation, and Direct Reprogramming. STAR Protoc..

[297] Afjeh-Dana E., Naserzadeh P., Moradi E., Hosseini N., Seifalian A.M., Ashtari B. (2022). Correction to: Stem Cell Differentiation into Cardiomyocytes: Current Methods and Emerging Approaches. Stem Cell Rev. Rep..

[298] Femminò S., Penna C., Margarita S., Comità S., Brizzi M.F., Pagliaro P. (2020). Extracellular Vesicles and Cardiovascular System: Biomarkers and Cardioprotective Effectors. Vascul. Pharmacol..

[299] Balbi C., Costa A., Barile L., Bollini S. (2020). Message in a Bottle: Upgrading Cardiac Repair into Rejuvenation. Cells.

[300] Pezhouman A., Nguyen N.B., Kay M., Kanjilal B., Noshadi I., Ardehali R. (2023). Cardiac Regeneration—Past Advancements, Current Challenges, and Future Directions. J. Mol. Cell. Cardiol..

[301] Zurek M., Johansson E., Palmer M., Albery T., Johansson K., Rydén-Markinhutha K., Wang Q.-D. (2020). Neuregulin-1 Induces Cardiac Hypertrophy and Impairs Cardiac Performance in Post–Myocardial Infarction Rats. Circulation.

[302] Täubel J., Hauke W., Rump S., Viereck J., Batkai S., Poetzsch J., Rode L., Weigt H., Genschel C., Lorch U. (2021). Novel Antisense Therapy Targeting MicroRNA-132 in Patients with Heart Failure: Results of a First-in-Human Phase 1b Randomized, Double-Blind, Placebo-Controlled Study. Eur. Heart J..

[303] Yaghoobi A., Rezaee M., Behnoush A.H., Khalaji A., Mafi A., Houjaghan A.K., Masoudkabir F., Pahlavan S. (2024). Role of Long Noncoding RNAs in Pathological Cardiac Remodeling after Myocardial Infarction: An Emerging Insight into Molecular Mechanisms and Therapeutic Potential. Biomed. Pharmacother..

[304] Omidian H., Babanejad N., Cubeddu L.X. (2023). Nanosystems in Cardiovascular Medicine: Advancements, Applications, and Future Perspectives. Pharmaceutics.

[305] Perveen S., Rossin D., Vitale E., Rosso R., Vanni R., Cristallini C., Rastaldo R., Giachino C. (2021). Therapeutic Acellular Scaffolds for Limiting Left Ventricular Remodelling-Current Status and Future Directions. Int. J. Mol. Sci..

[306] Cristallini C., Vitale E., Giachino C., Rastaldo R. (2020). Nanoengineering in Cardiac Regeneration: Looking Back and Going Forward. Nanomaterials.

[307] Kitsara M., Agbulut O., Kontziampasis D., Chen Y., Menasché P. (2017). Fibers for Hearts: A Critical Review on Electrospinning for Cardiac Tissue Engineering. Acta Biomater..

[308] Lin X., Liu Y., Bai A., Cai H., Bai Y., Jiang W., Yang H., Wang X., Yang L., Sun N. (2019). A Viscoelastic Adhesive Epicardial Patch for Treating Myocardial Infarction. Nat. Biomed. Eng..

[309] Chinyere I.R., Bradley P., Uhlorn J., Eason J., Mohran S., Repetti G.G., Daugherty S., Koevary J.W., Goldman S., Lancaster J.J. (2021). Epicardially Placed Bioengineered Cardiomyocyte Xenograft in Immune-Competent Rat Model of Heart Failure. Stem Cells Int..

